# Suppression of Pyruvate Dehydrogenase Kinase by Dichloroacetate in Cancer and Skeletal Muscle Cells Is Isoform Specific and Partially Independent of HIF-1α

**DOI:** 10.3390/ijms22168610

**Published:** 2021-08-10

**Authors:** Nives Škorja Milić, Klemen Dolinar, Katarina Miš, Urška Matkovič, Maruša Bizjak, Mojca Pavlin, Matej Podbregar, Sergej Pirkmajer

**Affiliations:** 1 Institute of Pathophysiology, Faculty of Medicine, University of Ljubljana, 1000 Ljubljana, Slovenia; nives.skorja@mf.uni-lj.si (N.Š.M.); klemen.dolinar@mf.uni-lj.si (K.D.); katarina.mis@mf.uni-lj.si (K.M.); umatkovic@onko-i.si (U.M.); matej.podbregar@mf.uni-lj.si (M.P.); 2 Institute of Anatomy, Faculty of Medicine, University of Ljubljana, 1000 Ljubljana, Slovenia; 3 Group for Nano and Biotechnological Applications, Faculty of Electrical Engineering, University of Ljubljana, 1000 Ljubljana, Slovenia; marusa.bizjak@ffa.uni-lj.si (M.B.); mojca.pavlin@fe.uni-lj.si (M.P.); 4 Pharmacy Institute, Faculty of Pharmacy, University of Ljubljana, 1000 Ljubljana, Slovenia; 5 Institute of Biophysics, Faculty of Medicine, University of Ljubljana, 1000 Ljubljana, Slovenia; 6 Department of Internal Medicine, Faculty of Medicine, University of Ljubljana, 1000 Ljubljana, Slovenia; 7 Department for Internal Intensive Care, General and Teaching Hospital Celje, 3000 Celje, Slovenia

**Keywords:** dichloroacetate, pyruvate dehydrogenase kinase, pyruvate dehydrogenase complex, cancer, skeletal muscle cells, myotubes

## Abstract

Inhibition of pyruvate dehydrogenase kinase (PDK) emerged as a potential strategy for treatment of cancer and metabolic disorders. Dichloroacetate (DCA), a prototypical PDK inhibitor, reduces the abundance of some PDK isoenzymes. However, the underlying mechanisms are not fully characterized and may differ across cell types. We determined that DCA reduced the abundance of PDK1 in breast (MDA-MB-231) and prostate (PC-3) cancer cells, while it suppressed both PDK1 and PDK2 in skeletal muscle cells (L6 myotubes). The DCA-induced PDK1 suppression was partially dependent on hypoxia-inducible factor-1α (HIF-1α), a transcriptional regulator of PDK1, in cancer cells but not in L6 myotubes. However, the DCA-induced alterations in the mRNA and the protein levels of PDK1 and/or PDK2 did not always occur in parallel, implicating a role for post-transcriptional mechanisms. DCA did not inhibit the mTOR signaling, while inhibitors of the proteasome or gene silencing of mitochondrial proteases CLPP and AFG3L2 did not prevent the DCA-induced reduction of the PDK1 protein levels. Collectively, our results suggest that DCA reduces the abundance of PDK in an isoform-dependent manner via transcriptional and post-transcriptional mechanisms. Differential response of PDK isoenzymes to DCA might be important for its pharmacological effects in different types of cells.

## 1. Introduction

Pyruvate dehydrogenase complex (PDC) is a multienzyme complex that serves as a gatekeeper between glycolysis and oxidation of glucose in the tricarboxylic acid cycle [1]. PDC comprises pyruvate dehydrogenase (E1), dihydrolipoamide acetyltransferase (E2), and dihydrolipoamide dehydrogenase (E3), which convert pyruvate to acetyl-CoA, as well as pyruvate dehydrogenase kinase (PDK) and pyruvate dehydrogenase phosphatase, which regulate phosphorylation of the α-subunit of E1 (PDHE1α). PDK phosphorylates PDHE1α, thereby inhibiting PDC and redirecting metabolism of pyruvate into lactate [2]. PDK has four isoforms (PDK1–4), which have distinct biochemical properties and tissue-specific expression patterns and functions [3,4,5,6]. Altered PDK function, such as inappropriate inhibition of PDC by PDKs, is associated with various pathologies, including cancer [7,8,9,10,11,12,13,14], insulin resistance and type 2 diabetes [14,15,16,17,18,19,20], sepsis [21], as well as pulmonary arterial hypertension [22]. Pharmacological inhibition of PDKs has therefore emerged as an attractive option for therapy of such disorders [14,20,23,24].

PDK1 is the only isoenzyme capable of phosphorylating all three inhibitory sites (site 1 [Ser293], site 2 [Ser300], and site 3 [Ser232]) of PDHE1α [25]. PDK1 incorporates more phosphate into PDC than other isoenzymes [26], while the rate of dephosphorylation is the lowest when PDC is phosphorylated by PDK1 (PDK2 > PDK4 >> PDK3 > PDK1) [26]. In the skeletal muscle of endurance athletes, the abundance of PDK1 is reduced, while gene silencing of PDK1 increased the activity of citrate synthase in C2C12 skeletal muscle cells [27]. In addition, PDK1 seems to have a major role in tumorigenicity [28]. All this indicates that PDK1 has important biological functions despite comparatively low expression levels in most tissues [3].

Dichloroacetate (DCA), a pyruvate analogue, inhibits PDK, thereby reducing the phosphorylation of PDHE1α, activating PDC, and shifting metabolism from glycolysis to glucose oxidation [29]. While PDK1 is less sensitive to DCA than PDK2 or PDK4 (PDK2 > PDK4 > PDK1 > PDK3) [3], DCA reduced the abundance of PDK1 in cancer cells [12,30] and fibroblasts [31], indicating that inhibition is not the only mechanism by which DCA reduces the PDK1 function. Upregulation of PDK1 by hypoxia-inducible factor-1 (HIF-1), a heterodimeric transcription factor comprising an oxygen-sensitive subunit HIF-1α and a constitutive subunit HIF-1β (aka aryl hydrocarbon receptor nuclear translocator (ARNT)) [32,33,34,35,36], suppresses mitochondrial respiration under hypoxic conditions [37,38]. DCA promotes the degradation of HIF-1α, which in turn lowers the expression of *PDK1* [39,40], suggesting a mechanism whereby DCA reduces the abundance of PDK1 by suppressing its transcription. However, DCA reduced the abundance of PDK1 protein in glioblastoma cells in one hour [30], implicating a role for the post-transcriptional mechanisms, such as regulation of the translation by the mammalian target of rapamycin (mTOR) or proteolysis.

Although PDK isoenzymes are subjected to distinct regulatory mechanisms [3,41,42], whether DCA reduces the protein abundance of all PDK isoenzymes independently of cell type has not been systematically investigated. The mechanism by which DCA reduces the abundance of PDK1 has also not been thoroughly characterized. Here, we examined whether DCA, a prototypical PDK inhibitor, altered the mRNA expression and the protein abundance of PDK isoenzymes in breast cancer (MDA-MB-231), prostate cancer (PC-3), and rat skeletal muscle cells (L6 myotubes). We found that DCA suppressed PDK isoenzymes in an isoform-dependent manner. Moreover, the suppression of PDK1 was partially independent of HIF-1α, which supports the idea that post-transcriptional mechanisms contribute to the effects of DCA on PDK1.

## 2. Results

### 2.1. DCA Markedly Reduces the Abundance of PDK1 in MDA-MB-231 and PC-3 Cancer Cells

**Figure 1:**
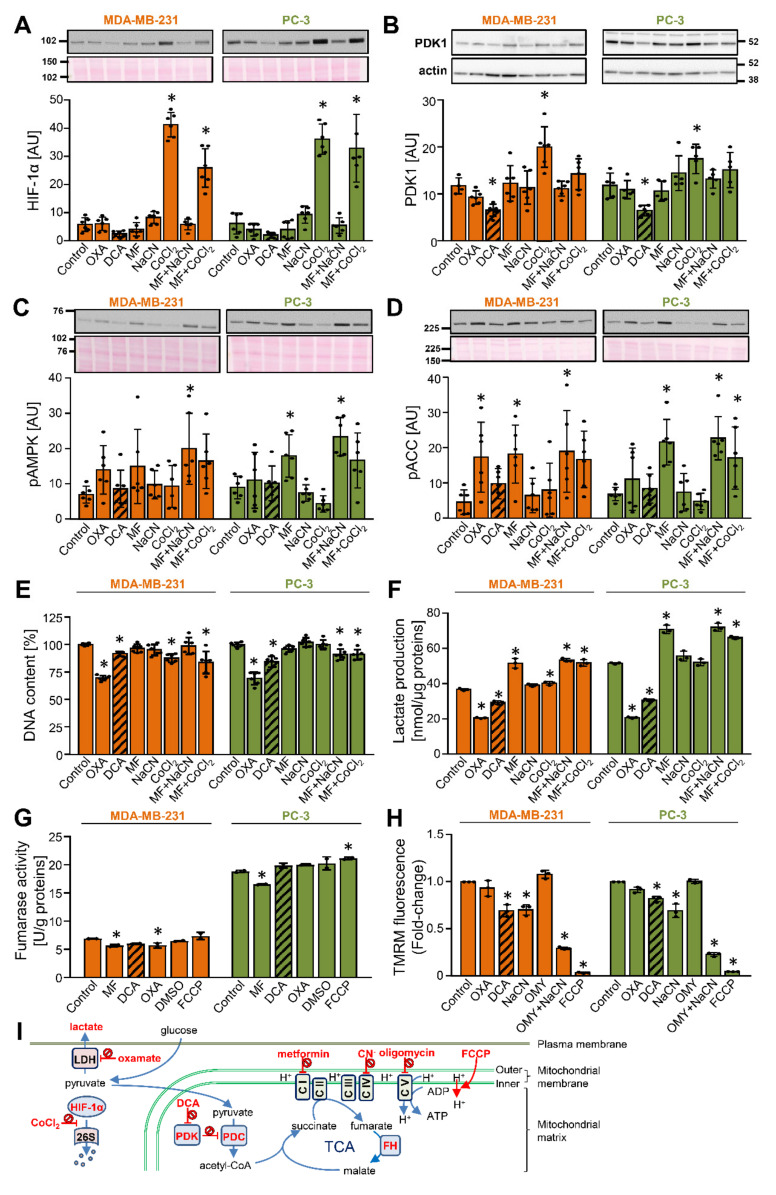
DCA reduces the abundance of PDK1 in MDA-MB-231 and PC-3 cancer cells. (**A**–**G**) Cells were treated with 10 mM oxamate (OXA), 10 mM DCA, 5 mM metformin (MF), 5 mM NaCN, 250 μM CoCl_2_, metformin + NaCN or metformin + CoCl_2_, or 0.5 μM FCCP or dimethyl sulfoxide (DMSO) for 24 h in RPMI medium without FBS (**A**–**D**,**F**) or with 10% FBS (**E**,**G**). Immunoblotting was used to evaluate the protein levels of (**A**) HIF-1α, (**B**) PDK1 and β-actin, (**C**) phospho-AMPKα^Thr172^ (pAMPK), and (**D**) phospho-ACC^Ser79^ (pACC). Total protein loading was evaluated by staining with Ponceau S (shown below the blots). Numbers next to the blots and Ponceau stains indicate molecular weight markers in kDa. (**E**) The total DNA content was estimated by Hoechst 33342 staining. (**A**–**E**) Results are means ± SD of two experiments in triplicate for each cell line (*n* = 6). (**F**) Lactate production in 24 h. Results are means ± SD of one experiment in triplicate (*n* = 3). (**G**) Activity of fumarase (fumarate hydratase). Results are means ± SD of one experiment in duplicate (*n* = 2). (**H**) MDA-MB-231 and PC-3 cancer cells were treated for 40 min with 10 mM oxamate (OXA), 10 mM DCA, 10 mM NaCN, 5 μM oligomycin (OMY), OMY + NaCN, or 40 μM FCCP in PBS with 1 g/L glucose 10 mM HEPES, 2 mM glutamine, and 1% FBS. Mitochondrial membrane potential was determined using TMRM. Results are means ± SD of three independent experiments (*n* = 3). (**I**) Site of action of compounds used in these experiments. Abbreviations: CI–V: mitochondrial complexes I–V, FH: fumarate hydratase (fumarase), PDC: pyruvate dehydrogenase complex, PDK: pyruvate dehydrogenase kinase, LDH: lactate dehydrogenase, 26S: the 26S proteasome, OMY: oligomycin; TCA: the tricarboxylic acid (Krebs) cycle. * *p* < 0.05 vs. Control.

To determine the responsiveness of PDK1 to DCA and other modulators of glycolysis or mitochondrial respiration, MDA-MB-231 and PC-3 cells were treated with 10 mM DCA as well as 10 mM oxamate (inhibitor of lactate dehydrogenase) [43], 5 mM metformin (inhibitor of complex I of mitochondrial respiratory chain [44,45]), 5 mM NaCN (inhibitor of complex IV), 250 μM CoCl_2_ (inhibitor of HIF-1α degradation [34,46]), or metformin in combination with NaCN or CoCl_2_ (the site of action of these compounds is schematically presented in Figure 1I). Treatment with 250 μM CoCl_2_, which was chosen based on our work on HIF-1α in cultured skeletal muscle cells [47,48,49], markedly increased the protein abundance of HIF-1α (Figure 1A) and PDK1 (Figure 1B). Conversely, DCA tended to reduce the protein levels of HIF-1α and PDK1 (Figure 1A,B). Other compounds did not alter HIF-1α or PDK1 levels, indicating that pharmacological modulation of the mitochondrial function or glycolysis per se is not sufficient to alter the PDK1 levels.

To assess the cellular energy status, activation of AMP-activated protein kinase (AMPK), a cellular energy sensor, was estimated by measuring the phosphorylation of AMPK at Thr172 (Figure 1C) and/or the phosphorylation of its substrate acetyl-CoA carboxylase (ACC) at Ser79 (Figure 1D). Metformin with or without other treatments increased or tended to increase the phosphorylation of AMPK and ACC, while other treatments did not alter the phosphorylation of AMPK and ACC significantly. These results indicated that oxidative metabolism is not only active in MDA-MB-231 and PC-3 cells, but that its inhibition at complex I causes energy stress and AMPK activation. Despite AMPK activation, which is considered to exert anti-proliferative effects, metformin did not reduce the total DNA content (Figure 1E), which indicated that proliferation was not suppressed [50]. Oxamate and DCA reduced the DNA content by ~25% and ~10–20% (Figure 1E), respectively, while other treatments had a similar or no effect.

The concentrations of lactate in cell medium were increased by metformin and decreased by DCA and oxamate (Figure 1F), consistent with previously published data and their established role as inhibitors of complex I [44,45,51,52], PDK [53,54,55,56], and lactate dehydrogenase [43,57,58] (sites of action are summarized in Figure 1I), respectively. Oxamate increased the phosphorylation of ACC, an indirect marker of energy stress, in MDA-MB-231 cells but not in PC-3 cells (Figure 1D), suggesting MDA-MB-231 cells are relatively more dependent on glycolytic ATP production than PC-3 cells. To explore this possible difference, cells were treated with DCA, metformin, oxamate, or mitochondrial uncoupler trifluoromethoxy carbonylcyanide phenylhydrazone (FCCP), which dissipates the proton gradient across the inner mitochondrial membrane (Figure 1I), for 24 h. The basal activity of fumarase (fumarate hydratase), an enzyme of the Krebs cycle, was markedly higher in PC-3 cells than in MDA-MB-231 cells (Figure 1G). DCA did not alter the fumarase activity, while metformin had a minor inhibitory effect. FCCP slightly increased the fumarase activity in PC-3 cells but had no effect on MDA-MB-231 cells. Taken together, these results were consistent with the notion that PC-3 cells are relatively more oxidative than MDA-MB-231 cells. Finally, as estimated by measuring the tetramethylrhodamine methyl ester (TMRM) fluorescence (Figure 1H), DCA reduced the mitochondrial potential in both cell lines, while oxamate had no significant effect.

**Figure 2:**
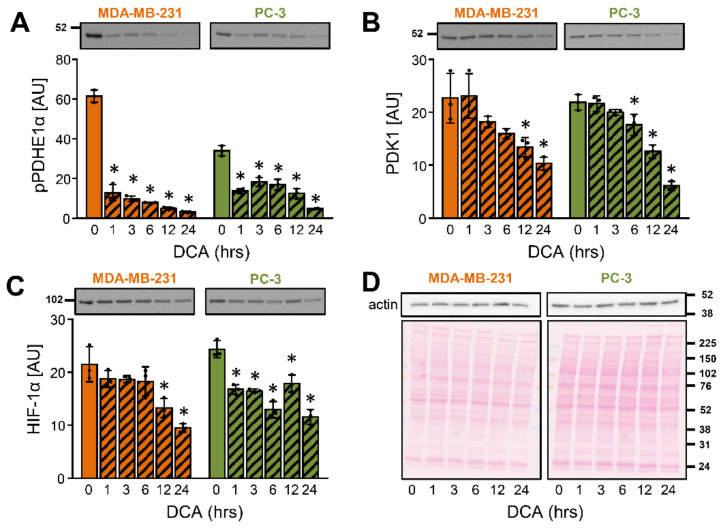
Time-dependent effects of DCA on the PDK1 and HIF-1α in MDA-MB-231 and PC-3 cancer cells. (**A**–**D**) Cells were treated with 10 mM DCA in serum-free RPMI medium for 24 h. Protein levels of (**A**) phospho-PDHE1α^Ser293^ (pPDHE1α), (**B**) PDK1, (**C**) HIF-1α, and (**D**) β-actin were determined with immunoblotting. (**D**) Ponceau S staining was used for the assessment of protein loading. Numbers next to the blots and Ponceau stains indicate molecular weight markers in kDa. Results are means ± SD of one experiment performed in triplicate for each cell line (*n* = 3). * *p* < 0.05 vs. 0 h.

To establish the time-course of DCA actions on PDK1, MDA-MB-231 and PC-3 cells were treated with 10 mM DCA for 1–24 h (Figure 2A–D). The phosphorylation of PDHE1α at site 1 [Ser293] is catalyzed by all PDK isoenzymes [26] and was therefore used as an indirect indicator of the PDK inhibition by DCA. The phosphorylation of PDHE1α was reduced within 1 h of treatment with DCA in both cancer cell lines (Figure 2A), consistent with an acute inhibition of PDK isoenzymes. The abundance of PDK1 was significantly reduced after 12 h in MDA-MB-231 cells and after 6 h in PC-3 cells (Figure 2B). The progressive suppression of PDK1 was preceded by a reduction of the HIF-1α protein levels (Figure 2C), which is compatible with the idea that DCA regulated the abundance of PDK1 via HIF-1α.

### 2.2. DCA Reduces the Abundance of PDK1 in MDA-MB-231 and PC-3 Cancer Cells

**Figure 3:**
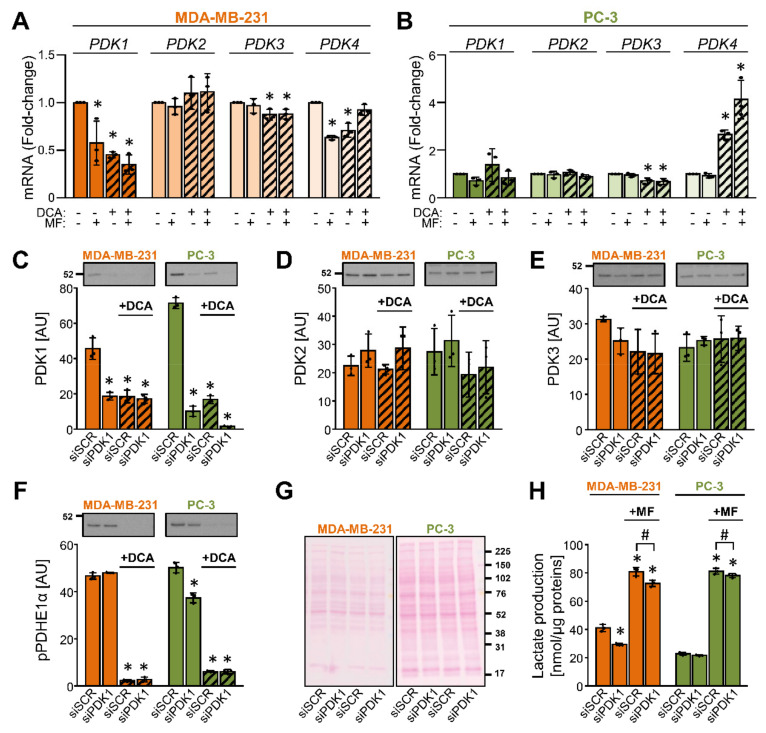
DCA reduces the abundance of PDK1 in MDA-MB-231 and PC-3 cells. (**A**,**B**) Cancer cells were treated with 10 mM DCA and/or 5 mM metformin (MF) in serum-free RPMI medium for 24 h. The mRNA expression of PDK1–4 isoenzymes was evaluated in (**A**) MDA-MB-231 and (**B**) PC-3 cells with qPCR (endogenous control: cyclophilin). (**C**–**F**) After treating MDA-MB-231 and PC-3 cells with the PDK1 siRNA (siPDK1) or the scrambled siRNA (siSCR), cells were exposed to 10 mM DCA for 24 h in serum-free RPMI medium. Immunoblotting was used to evaluate the protein levels of (**C**) PDK1, (**D**) PDK2, (**E**) PDK3, and (**F**) phospho-PDHE1α^Ser293^ (pPDHE1α). (**G**) Ponceau S staining was used to evaluate the total protein loading. Numbers next to the blots and Ponceau stains indicate molecular weight markers in kDa. (**H**) Lactate production in PDK1-deficient (siPDK1) and control (siSCR) cells treated with 5 mM metformin (MF) for 24 h. Results are means ± SD of one experiment performed in triplicate for each cell line (*n* = 3). * *p* < 0.05 vs. untreated cells (**A**,**B**) or siSCR (**C**–**F**,**H**) and # *p* < 0.05 as indicated.

PDK isoenzymes (PDK1–4) have tissue-specific expression patterns and functions [3]. To test how DCA affected the gene expression of the four PDK isoenzymes, MDA-MB-231 and PC-3 cells were treated with 10 mM DCA for 24 h (Figure 3A,B). Metformin (5 mM) was used as a comparison. In MDA-MB-231 cells, DCA and/or metformin reduced the expression of PDK1, PDK3, and PDK4 mRNA, while the expression of PDK2 mRNA was unaltered (Figure 3A). In PC-3 cells, DCA did not alter the expression of PDK1 and PDK2 mRNA, but it reduced the expression of PDK3 mRNA and increased the expression of PDK4 mRNA (Figure 3B). Metformin alone had no effect on the expression of PDK isoenzymes in PC-3 cells (Figure 3B). These results indicated that the effects of DCA and metformin on gene expression differed between PDK isoenzymes as well as MDA-MB-231 and PC-3 cells. 

To determine the effect of DCA on the abundance of PDK isoenzymes and to test the specificity of primary antibodies, MDA-MB-231 and PC-3 cells were treated with 10 nM siRNA for PDK1 (siPDK1) or 10 nM non-targeting (scrambled) siRNA (siSCR) (Figure 3C–H). After 24 h, growth medium was removed, and cells were treated with 10 mM DCA in serum-free RPMI medium for the next 24 h (Figure 3C–H). Gene silencing of PDK1 markedly reduced the protein abundance of PDK1 (Figure 3C) but did not significantly affect the protein levels of PDK2 (Figure 3D) and PDK3 (Figure 3E). Due to multiple apparently non-specific bands, we were unable to reliably assess the abundance of PDK4. Treatment with DCA reduced the PDK1 protein levels in the control and the PDK1 knock-down cells but had no or a modest statistically non-significant effect on PDK2 or PDK3. The phosphorylation of PDHE1α at Ser293 was markedly suppressed by the DCA treatment (Figure 3F). Silencing of PDK1 had a minor effect on the phosphorylation of PDHE1α in PC-3 cells but had no effect in MDA-MB-231 cells (Figure 3F). PDK1-deficient cells, especially MDA-MB-231 cells, tended to have lower lactate production (Figure 3H).

### 2.3. The Suppression of PDK1 by DCA Is Partially Independent of the Transcriptional Control via HIF-1α

**Figure 4:**
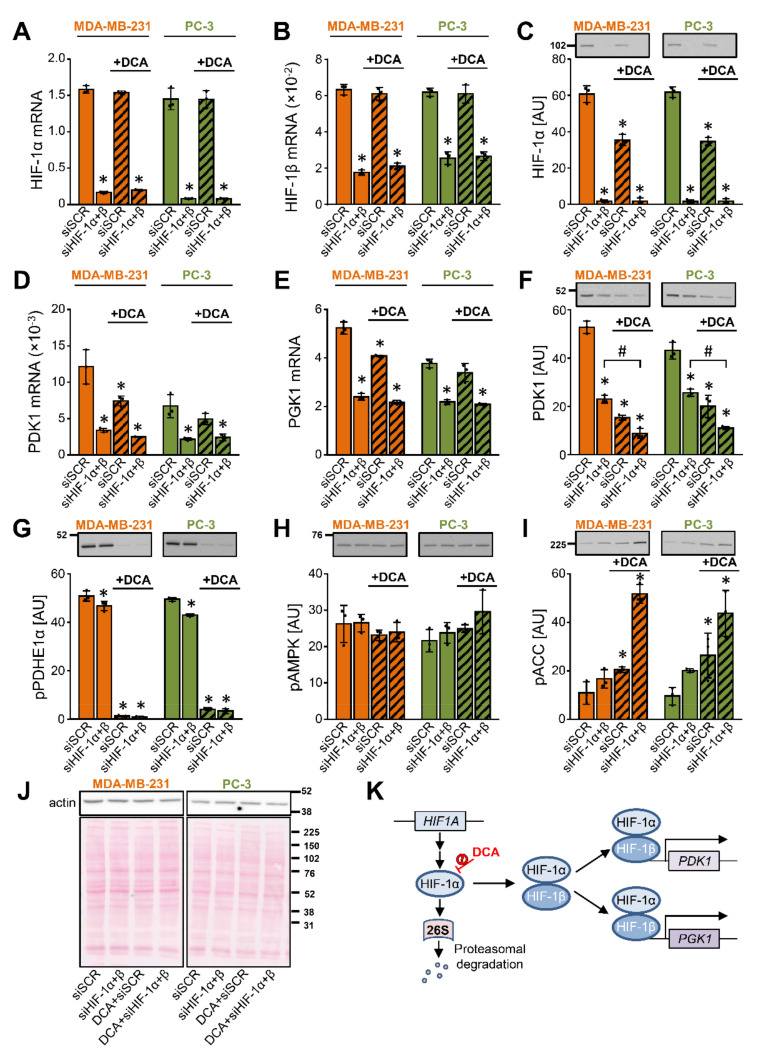
The suppression of PDK1 by DCA is partially independent of the transcriptional control via HIF-1α. (**A–J**) MDA-MB-231 and PC-3 cells were transfected with siRNAs against HIF-1α and HIF-1β to produce a double knock-down (siHIF-1α + β). They were subsequently treated with 10 mM DCA for 24 h in serum-free RPMI medium. The mRNA expression of (**A**) HIF-1α, (**B**) HIF-1β, (**D**) PDK1, and (**E**) PGK1 was estimated by qPCR (endogenous control: cyclophilin). The protein levels of (**C**) HIF-1α, (**F**) PDK1, (**G**) phospho-PDHE1α^Ser293^ (pPDHE1α), (**H**) phospho-AMPKα^Thr172^ (pAMPK), (**I**) phospho-ACC^Ser79^ (pACC), and (**J**) β-actin were determined with immunoblotting. (**J**) Protein loading was evaluated by the Ponceau S staining. Numbers next to the blots and Ponceau stains indicate molecular weight markers in kDa. (**K**) Schematic representation of regulation of gene expression by HIF-1. Results are means ± SD of one experiment performed in triplicate for each cell line (*n* = 3). * *p* < 0.05 vs. siSCR and # *p* < 0.05 as indicated.

To determine whether the DCA-induced suppression of PDK1 in MDA-MB-231 and PC-3 cells is HIF-1α dependent, we knocked down HIF-1α and HIF-1β (aka ARNT) (Figure 4A–J), which together form the functional HIF-1 heterodimer (HIF-1α/HIF-1β) (Figure 4K). Treatment with 10 mM DCA for 24 h had no effect on the HIF-1 mRNA expression (Figure 4A,B), but it reduced the HIF-1α protein content in both cancer cell lines (Figure 4C), indicating HIF-1α was regulated by DCA on a post-transcriptional level.

Gene silencing of HIF-1 reduced the mRNA expression of PDK1 (Figure 4D) and phosphoglycerate kinase 1 (PGK1) (Figure 4E), a glycolytic enzyme and a target of HIF-1α [59] (Figure 4K), indicating HIF-1 drives their expression under basal conditions. DCA had no additional effect on the PDK1 and PGK1 mRNA levels in the HIF-1-deficient cells. Nevertheless, DCA reduced the protein abundance of PDK1 in the control and HIF-1-deficient cells (Figure 4F). Taken together, the discrepancy between the mRNA and protein responses indicated that regulation of the PDK1 protein abundance by DCA was partially independent of the transcriptional control by HIF-1α.

The silencing of HIF-1 had only a minor effect on the phosphorylation of PDHE1α (Ser293), while DCA almost completely dephosphorylated it (Figure 4G). In the control cells, DCA increased the inhibitory phosphorylation of ACC (Ser79) without altering the phosphorylation of AMPK (Thr172) (Figure 4H,I). The DCA-stimulated phosphorylation of ACC was even more pronounced in the HIF-1-deficient cells (Figure 4I), indicating that gene silencing of HIF-1α and HIF-1β combined with the DCA treatment led to a pronounced inhibition of ACC.

### 2.4. Inhibition of the mTOR Pathway Does Not Contribute to the DCA-Induced Suppression of PDK1

**Figure 5:**
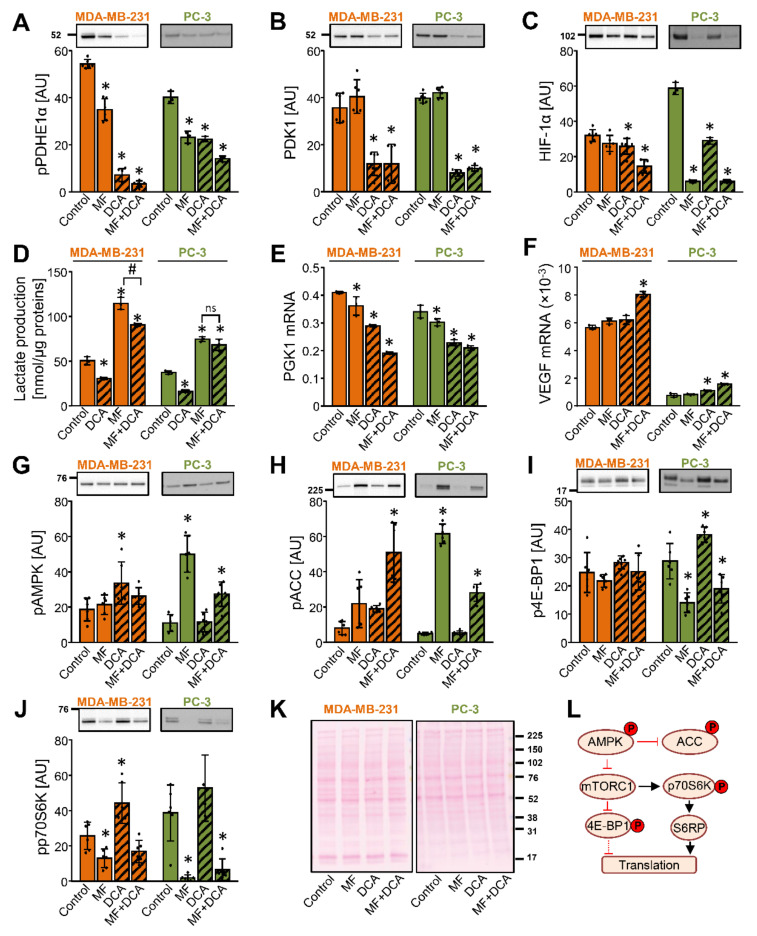
Inhibition of the mTOR pathway does not contribute to the DCA-induced suppression of PDK1. (**A**–**K**) MDA-MB-231 and PC-3 cells were treated with 5 mM metformin (MF), 10 mM DCA, or both in serum-free RPMI medium for 24 h. The protein level of (**A**) phospho-PDHE1α^Ser293^ (pPDHE1α), (**B**) PDK1, (**C**) HIF-1α, (**G**) phospho-AMPKα^Thr172^ (pAMPK), (**H**) phospho-ACC^Ser79^ (pACC), (**I**) phospho-4E-BP1^Thr 37/46^ (p4E-BP1), and (**J**) phospho-p70S6K^Thr389^ was determined with immunoblotting. (**K**) Total protein loading was evaluated with the Ponceau S staining. Numbers next to the blots and Ponceau stains indicate molecular weight markers in kDa. Results are means ± SD of two experiments performed in triplicate (*n* = 6). * *p* < 0.05 vs. Control. (**D**) Lactate production in MDA-MB-231 and PC-3 cells treated with 10 mM DCA and/or 5 mM metformin for 24 h. The mRNA expression of (**E**) PGK1 and (**F**) VEGF was measured with qPCR (endogenous control: cyclophilin). (**D**–**F**) Results are means ± SD of one experiment performed in triplicate for each cell line (*n* = 3). * *p* < 0.05 vs. Control and # *p* < 0.05 as indicated (**L**) Schematic overview of the AMPK and mTOR signaling. Phosphoproteins that were evaluated in this study are indicated with P in a red circle. S6RP: S6 ribosomal protein. For other abbreviations, see the text.

The activation of AMPK inhibits the mammalian target of rapamycin complex 1 (mTORC1) signaling [60] (schematic overview in Figure 5L), which may lead to the suppression of protein synthesis [61,62], including down-regulation of HIF-1α [63]. The DCA-induced increase in the phosphorylation of ACC suggested a possible involvement of AMPK and mTORC1 in regulation of the PDK1 protein levels (Figure 4I). To explore this possibility in more detail, MDA-MB-231 and PC-3 cells were treated with 10 mM DCA and 5 mM metformin for 24 h (Figure 5).

DCA and/or metformin reduced the phosphorylation of PDHE1α at Ser293 (Figure 5A), but only DCA reduced the abundance of PDK1 (Figure 5B). Metformin potently increased the lactate production even in the presence of DCA (Figure 5D), suggesting that inhibition of complex I can override the DCA-induced activation of PDC. While the HIF-1α mRNA expression was unaltered by the metformin treatment and increased by the DCA or the combined metformin and DCA treatment (Supplementary Materials Figure S1), the protein abundance of HIF-1α was reduced by all treatments in PC-3 cells (Figure 5C).

Despite the marked reduction of HIF-1α by metformin, the protein abundance of PDK1 was reduced only in the presence of DCA (Figure 5B). Similarly, while the combined treatment with metformin and DCA was a particularly potent suppressor of HIF-1α in MDA-MB-231 cells (Figure 5C), DCA alone was sufficient to reduce the protein levels of PDK1 (Figure 5B). DCA and metformin reduced the expression of PGK1 in MDA-MB-231 and PC-3 cells (Figure 5E). Conversely, the expression of vascular endothelial growth factor (VEGF), also a target gene of HIF-1α, was increased by a co-treatment with DCA and metformin (Figure 5F). Taken together, these results showed that a reduction of the HIF-1α protein levels does not lead to uniform suppression of its downstream target genes. Moreover, they again indicated that regulation of the PDK1 protein levels is partially independent of HIF-1α, implicating a role for the post-transcriptional regulatory mechanisms.

In PC-3 cells, metformin markedly increased the phosphorylation of AMPK (Figure 5G) and ACC (Figure 5H). The AMPK activation was paralleled by a reduced phosphorylation of eukaryotic translation initiation factor 4E-binding protein 1 (4E-BP1) at Thr37/46 (Figure 5I) and p70 ribosomal protein S6 kinase (p70S6K) at Thr389 (Figure 5J), indicating that metformin inhibited the mTORC1 pathway [64] (Figure 5L). The combined treatment with metformin and DCA had a similar effect. In MDA-MB-231 cells, metformin non-significantly increased the phosphorylation of ACC and reduced the phosphorylation of p70S6K, while it did not alter the phosphorylation of AMPK and 4E-BP1, indicating MDA-MB-231 cells were less responsive to metformin, as we had previously observed [50,65,66]. DCA alone did not reduce the phosphorylation of 4E-BP1 or p70S6K neither in MDA-MB-231 or PC-3 cells. Taken together, these results provided evidence to suggest that inhibition of the mTOR signaling did not contribute to the suppression of PDK1 by DCA. Further, as evident from the effects of metformin, inhibition of mTOR signaling does not necessarily lead to a reduction in the PDK1 protein levels.

### 2.5. Inhibition of the Proteasome or Mitochondrial Proteases Does Not Prevent DCA-Induced Suppression of PDK1 in Cancer Cells

Since a reduction in the PDK1 protein levels did not seem to require repression of the *PDK1* gene (Figure 4) or inhibition of translation via inactivation of the mTOR pathway (Figure 5), we tested the possibility that DCA promoted the proteolysis of PDK1. MDA-MB-231 and PC-3 cells were treated with the proteasome inhibitor MG-132 for 1–12 h, which increased the protein abundance of HIF-1α in MDA-MB-231 and PC-3 cells within 3 h and 1 h, respectively (Supplementary Materials Figure S2A), indicating effective inhibition of the proteasome. In contrast to HIF-1α, the protein levels of PDK1 were decreased by MG-132 (Supplementary Materials Figure S2B), while the phosphorylation of PDHE1α at Ser293 was transiently increased (Supplementary Materials Figure S2C).

**Figure 6:**
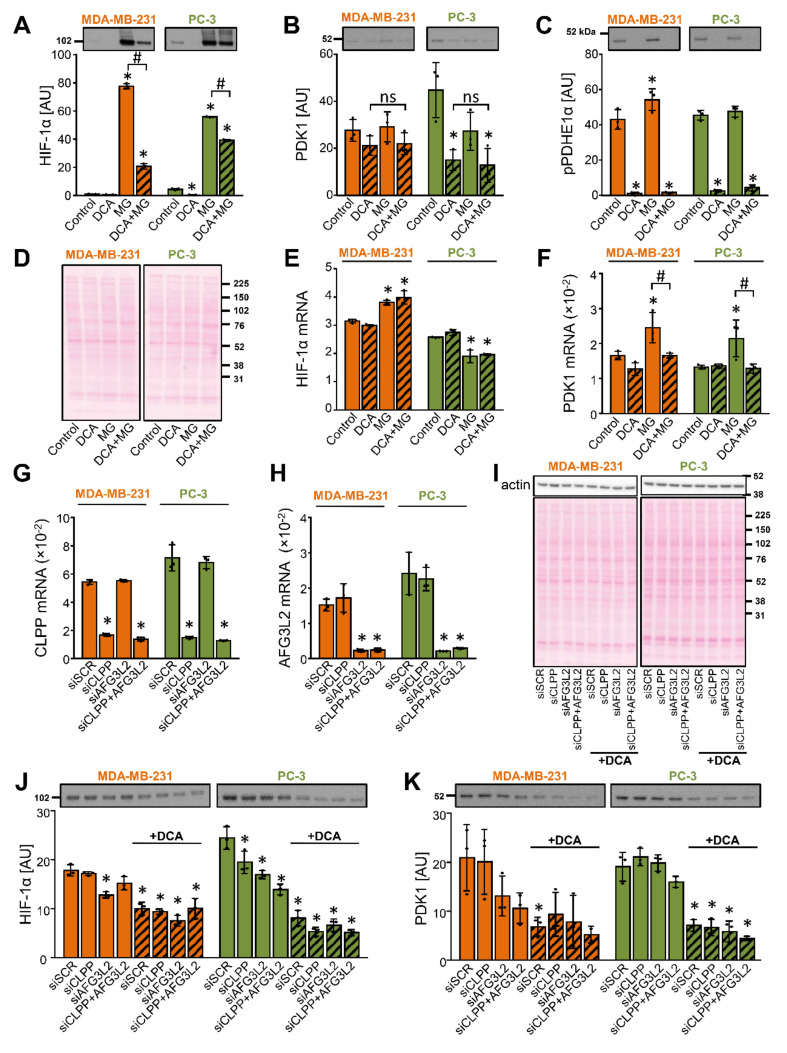
Inhibition of the proteasome or mitochondrial proteases does not prevent the DCA-induced suppression of PDK1 in MDA-MB-231 and PC-3 cells. (**A**–**F**) Cells were treated with DCA (10 mM) for 12 h in serum-free RPMI medium. MG-132 (10 μM, MG) was added during the final 6 h. (**G**–**K**) Cells were transfected with 20 nM scrambled siRNA (siSCR) or 10 nM CLPP and/or AFG3L2 siRNA (siCLPP and siAFG3L2). (**G**,**H**) Gene expression was assessed 48 h after the transfection. (**J**,**K**) Cells deficient in CLPP (siCLPP) and/or AFG3L2 (siAFG3L2) and control (siSCR) cells were treated with 10 mM DCA in serum-free RPMI medium for 24 h. The mRNA expression of (**E**) HIF-1α, (**F**) PDK1, (**G**) CLPP, and (**H**) AFG3L2 was estimated with qPCR (endogenous control: cyclophilin). The abundance of (**A**,**J**) HIF-1α, (**B**,**K**) PDK1, (**C**) phospho-PDHE1α^Ser293^ (pPDHE1α), and (**I**) β-actin was determined with immunoblotting. (**D**,**I**) Protein loading was evaluated with the Ponceau S staining. Numbers next to the blots and Ponceau stains indicate molecular weight markers in kDa. Results are means ± SD of one experiment, performed in triplicate, for each cell line and condition (*n* = 3). * *p* < 0.05 vs. Control or siSCR and # *p* < 0.05 as indicated.

To evaluate if DCA stimulated the proteasomal degradation of PDK1, MDA-MB-231 and PC-3 cells were treated with 10 mM DCA for 12 h and MG-132 (10 μM) was added for the final 6 h. The treatment with MG-132 had divergent effects on the HIF-1α mRNA levels (Figure 6E), but it potently increased the protein levels of HIF-1α, which was opposed by DCA (Figure 6A). This result was consistent with the mechanism whereby DCA represses the *PDK1* gene by promoting HIF-1α degradation. In MDA-MB-231 cells, MG-132 did not significantly affect the abundance of PDK1 protein (Figure 6B) despite an increase in mRNA levels (Figure 6F). In PC-3 cells, the abundance of PDK1 was reduced by DCA in the presence or absence of MG-132 (Figure 6B), highlighting that DCA may reduce the PDK1 levels without suppressing the expression of *PDK1*. DCA markedly reduced the phosphorylation of PDHE1α with or without MG-132 (Figure 6C). A similar result was obtained with 300 nM MG-262, an inhibitor of both proteasome and the mitochondrial Lon protease, which itself reduced PDK1 protein levels, while failing to prevent the DCA-induced suppression of PDK1 (unpublished observations by N.Š.M. and S.P.). These results did not support the notion that DCA stimulated the proteasomal degradation of PDK1.

DCA promotes a mitochondrial calcium accumulation [67], while calpains, calcium-dependent cysteine proteases, were shown to be involved in the degradation of HIF-1α [68]. However, neither calpain inhibitor III nor a protease inhibitor cocktail, which contained 4-(2-aminoethyl)-benzenesulfonyl fluoride (AEBSF), aprotinin, bestatin, E-64, leupeptin and pepstatin A, prevented the DCA-induced reduction of the PDK1 protein levels (Supplementary Materials Figure S2D–G). To assess the role of the mitochondrial matrix proteases, we performed gene silencing of the caseinolytic mitochondrial matrix peptidase proteolytic subunit (CLPP), a component of the Clp protease complex CLPXP, and AFG3-like protein 2 (AFG3L2), a catalytic subunit of the matrix AAA protease (Figure 6G,H). DCA (10 mM) treatment was started 48 h after transfection with siRNAs. The knockdown of CLPP and/or AFG3L2 modestly decreased the protein level of HIF-1α (Figure 6J), while DCA significantly decreased the protein level of PDK1 (Figure 6K) despite gene silencing of CLPP and/or AFG3L2, which shows that these proteases are not involved in the suppression of PDK1 by DCA.

### 2.6. DCA Reduces the Abundance of PDK1 and PDK2 in L6 Myotubes despite Upregulation of HIF-1α and Inhibition of the Proteasome

**Figure 7:**
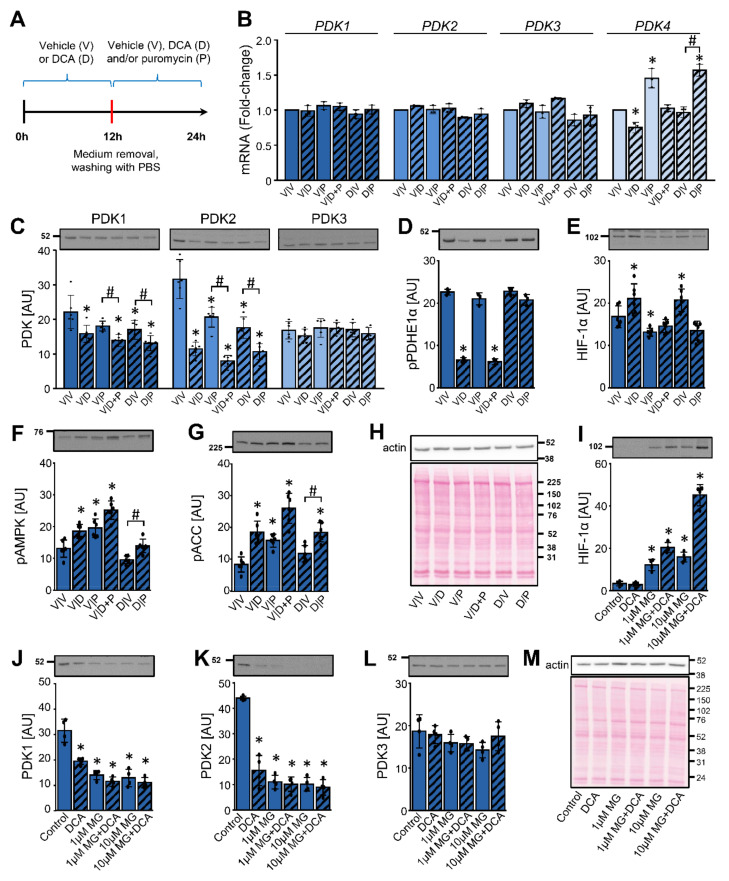
DCA reduces the abundance of PDK1 and PDK2 in L6 myotubes despite upregulation of HIF-1α and inhibition of the proteasome. (**A**–**H**) L6 myotubes were treated with 10 mM DCA or vehicle in serum-free minimal essential medium-α (MEMα). After 12 h of treatment, medium was removed, cells were washed with PBS and fresh MEMα− with vehicle (V), 10 mM DCA (D), and/or 0.5 μg/mL puromycin (P) was added for the next 12 h. (**B**) The gene expression levels of PDK1, PDK2, PDK3, and PDK4 were analyzed with qPCR (endogenous control: β-actin). Immunoblotting was used to estimate the protein abundance of (**C**) PDK1, PDK2, and PDK3, (**D**) phospho-PDHE1α^Ser293^ (pPDHE1α), (**E**) HIF-1α, (**F**) phospho-AMPK^Thr172^ (pAMPK), and (**G**) phospho-ACC^Ser79^ (pACC). (**H**) A representative Ponceau S staining of the membrane. Results are means ± SD of two independent experiments, each performed in three replicates (*n* = 6). *p < 0.05 vs. V/V and # *p* ≤ 0.05 as indicated. (**I**–**M**) The differentiated L6 cells (myotubes) were treated with MG-132 (1 or 10 μM MG) for 1 h, which was followed by a 24-h treatment with DCA (10 mM) and/or MG-132 (1 or 10 μM). Immunoblotting was used to estimate the abundance of (**I**) HIF-1α, (**J**) PDK1, (**K**) PDK2, and (**L**) PDK3. (**M**) Protein loading was evaluated with the Ponceau S staining. Numbers next to the blots and Ponceau stains indicate molecular weight markers in kDa. Results are means ± SD of one experiment in four replicates (*n* = 4). * *p* < 0.05 vs. Control and # *p <* 0.05 as indicated.

PDK1 is also regulated by HIF-1α in skeletal muscle, where it seems to play an important role in the adaptation to exercise or hypoxia [27,69]. We therefore assessed whether effects of DCA on PDK1 in the differentiated L6 skeletal muscle cells (myotubes) mimic those in cancer cells. L6 myotubes were treated with 10 mM DCA for 12 h, which was followed by a 12-h treatment with DCA (10 mM) and/or puromycin (0.5 μg/mL) (Figure 7A), an inhibitor of the translation.

Excepting modest alterations in the expression of PDK4 mRNA, the gene expression of PDK isoenzymes was stable throughout the experiment (Figure 7B). DCA reduced the protein level of PDK1 and PDK2, while PDK3 remained unaltered (Figure 7C). The abundance of PDK1 and especially PDK2 was lower in the myotubes treated with DCA and puromycin (V/D + P) than in the myotubes treated with puromycin (V/P) alone. A washout of DCA (D/V) enabled a partial recovery of the PDK1 and PDK2 protein levels, which was blocked if the DCA washout was followed by the puromycin treatment (D/P) (Figure 7C). DCA markedly dephosphorylated PDHE1α (V/D), but the phosphorylation was completely recovered after the DCA washout (D/V) despite a continued suppression of PDK1 and PDK2 (Figure 7D). In direct contrast to the results in MDA-MB-231 and PC-3 cells, DCA slightly increased the protein levels of HIF-1α in L6 myotubes with (D/V) or without (V/D) washout (Figure 7E). The DCA-induced increase in the HIF-1α levels was prevented by puromycin (V/D + P). As assessed by the phosphorylation of AMPK and ACC (Figure 7F,G), both DCA and puromycin activated AMPK, suggesting that L6 myotubes were under energy stress during these treatments.

To assess the role of the proteasome, L6 myotubes were pretreated with MG-132 (1 μM or 10 μM) for 1 h, which was followed by a 24-h treatment with or without MG-132 (1 μM or 10 μM) and/or DCA (10 mM) (Figure 7I–M). The protein levels of HIF-1α, which were very low under the basal conditions, were markedly upregulated by MG-132 (Figure 7I), which suggested effective inhibition of the proteasome. Combined treatment with DCA and MG-132 also increased the abundance of HIF-1α. DCA markedly decreased the protein levels of PDK1 (Figure 7J) and PDK2 (Figure 7K). The phosphorylation of PDHE1α (Ser293) in L6 cells was decreased by DCA as well as MG-132 (Supplementary Materials Figure S3A), which paralleled the loss of PDK1 and PDK2. MG-132 not only did not prevent the suppression of PDK1 and PDK2 by DCA, but it reduced the levels of both proteins (Figure 7J,K). The protein level of PDK3 remained unaltered during the DCA and/or MG-132 treatment (Figure 7L).

We also assessed the activating phosphorylation of PDK1 at Thr338, which is catalyzed by PGK1 [70]. DCA alone did not alter the phosphorylation of PDK1 (Supplementary Materials Figure S3B). During the treatment with MG-132, multiple immunoreactive bands appeared, which indicated a possible accumulation of the ubiquitinated phospho-PDK1. DCA increased the phosphorylation of AMPK and ACC, indicating AMPK activation (Supplementary Materials Figure S3C,D). Taken together, these results suggest that DCA reduced the protein levels of PDK1 and PDK2 despite inhibition of the proteasome. Moreover, this reduction seemed to be independent of HIF-1α and alterations in gene expression.

## 3. Discussion

Our study showed that DCA had an isoform-dependent effect on the mRNA expression and protein abundance of PDK isoenzymes in MDA-MB-231 and PC-3 cancer cells and L6 myotubes. The effects of DCA on the PDK mRNA expression did not always correlate with its effects on the PDK protein abundance, indicating that DCA exerted its effects on the transcriptional as well as the post-transcriptional levels. According to our results, the post-transcriptional regulation of PDK isoenzymes by DCA did not involve the mTOR pathway, the proteasome, or the mitochondrial proteases.

Among the PDK isoenzymes, PDK2 is the most and PDK3 is the least sensitive to the inhibition by DCA (PDK2 > PDK4 > PDK1 > PDK3) [3]. However, in MDA-MB-231 and PC-3 cells, DCA potently suppressed only PDK1, suggesting that the effects of DCA on the abundance of PDK isoenzymes did not correlate with their sensitivity to inhibition by DCA. This means that DCA can suppress the PDK-mediated phosphorylation of PDHE1α and thereby activate PDC via at least two mechanisms. The first mechanism is an acute inhibition of PDK, the degree of which would depend on the concentration of DCA and the sensitivity of PDK isoenzymes. A corollary to this is that tissues expressing more sensitive isoenzymes, such as PDK2 or PDK4, are more sensitive to PDK inhibition and PDC activation by DCA. However, we and others [12,30,31] have shown that DCA potently reduces the abundance of PDK1 in multiple cell types despite PDK1 being 5-fold less sensitive to the inhibition by DCA than PDK2 [3]. While alterations in the protein levels of enzymes, such as PDK, do not directly correlate with their enzymatic activity, a reduction in the total abundance of PDKs would reduce the capacity of the cell to inhibit PDC. The second mechanism by which DCA promotes PDC activation is therefore a reduction in the total cellular content of PDK, which would have an additional and more prolonged effect on PDC activity than a simple inhibition of PDK.

That DCA altered the protein levels of PDK isoenzymes in an isoform-specific manner has potentially at least two important consequences. Firstly, inhibition of enzyme activity is an acute phenomenon, whereas a reduction in protein levels requires protein resynthesis as demonstrated in experiments with puromycin-treated L6 myotubes. DCA would therefore be expected to acutely promote the PDC dephosphorylation (activation) due to inhibition of the total PDK activity, while suppressing the PDC phosphorylation (inactivation) also on a longer time scale due to reduced PDK abundance. However, while PDK isoenzymes that are most sensitive to inhibition by DCA would be expected to contribute most to the acute activation of PDC, even PDK isoenzymes that are less susceptible to inhibition by DCA could contribute to chronic stimulation of PDC if their abundance is reduced by it. Secondly, an isoform-dependent effect of DCA on the abundance of PDK isoenzymes suggests that DCA alters not only the total PDK protein levels, but also the ratios between the four isoenzymes. PDK isoenzymes differ in their responsiveness to acute physiological regulators of enzyme activity, such as pyruvate, NADH, and acetyl-CoA [3], as well as their transcriptional or translational responses to various acute or chronic physiological stimuli, such as fasting, exercise, or hypoxia [41,42,71,72]. Isoform-dependent alterations in the protein abundance of PDK isoenzymes therefore likely have a functional and not only quantitative effect on the regulation of PDC.

DCA (10 mM) reduced the abundance of PDK1 within 6 or 12 h in PC-3 and MDA-MB-231 cells, respectively. Interestingly, while a decrease in the abundance of PDK1 protein in response to the DCA treatment was often reported, the timeframe within which it was previously studied varied widely. For instance, glioblastoma cells were treated with 1 mM DCA for 1 h [30], retinoblastoma cells with 3 mM DCA for 48 h [12], and fibroblasts with 10 mM DCA for 72 h [31]. A slower gradual reduction of the PDK1 protein levels in response to DCA treatment would be consistent with the transcriptional or the translational regulation of PDK1, while a rapid decrease is suggestive of stimulation of proteolysis. Gene silencing of HIF-1 and the treatment with DCA both lowered the mRNA and protein levels of PDK1, while CoCl_2_, a potent inducer of HIF-1α, increased the protein levels of PDK1. While these results are consistent with the idea that DCA regulates PDK1 via the gene expression, it is important to note that the extent to which HIF-1 regulates the *PDK1* gene expression in normoxia likely depends on the cell type. Indeed, in cells that effectively lack HIF-1α in normoxia, the *PDK1* expression is dependent on HIF-1α only or primarily under hypoxic conditions [37,73].

Metformin lowered the protein levels of HIF-1α in PC-3 cells, consistent with previous observations [74]. However, despite the decrease in the HIF-1α levels, PDK1 remained unaltered, which again shows that the transcription is not the only site of regulation of PDK1. Despite having a different effect on PDK1 than DCA, metformin decreased the phosphorylation of PDHE1α, indicating activation of PDC. At the same time, metformin increased the lactate production, which was consistent with the inactivation of PDC. While this may seem to be a paradoxical result, discrepancies between alterations in the phosphorylation of PDHE1α and activity of PDC were noted before [75], indicating that the phosphorylation status of PDHE1α is not always a reliable measure of the PDC activity. The dephosphorylation of PDHE1α may perhaps indicate a feedback mechanism, whereby the inhibition of the mitochondrial respiratory chain at the complex I by metformin leads to activation of the PDC as a compensatory mechanism. However, in contrast to what we observed, metformin increased the phosphorylation of PDHE1α (Ser293) and lactate production [76], while the metformin treatment in mice had no effect on the phosphorylation of PDHE1α at Ser293 or Ser300 [77]. Divergent results suggest that the effects of metformin on the phosphorylation status of PDHE1α are context or cell type dependent.

Alterations in the PDK1 mRNA and protein levels were not always in correlation, which was especially evident in PC-3 cancer cells and L6 myotubes. These discrepancies indicate that DCA regulates PDK1 also through the post-transcriptional mechanisms. For instance, inhibition of the mTOR pathway could block the translation of PDK1 mRNA, which would be consistent with observations that DCA inhibits the mTOR signaling pathway [78,79]. However, as assessed by the phosphorylation of 4E-BP1 and p70S6K, two of the downstream effectors of mTORC1 [64], DCA did not suppress the mTORC1 signaling in MDA-MB-231 and PC-3 cells. Furthermore, metformin, which suppressed the mTORC1 signaling in these cells, had no effect on the PDK1 protein levels. Taken together, our results do not support the idea that DCA reduced the PDK1 protein abundance in MDA-MB-231 or PC-3 cells by inhibiting the PDK1 translation via the mTORC1 signaling pathway and suggest the involvement of other post-transcriptional mechanisms, such as protein degradation.

PDK1 is ubiquitinated and degraded in the proteasome [28], while DCA reduces the protein abundance of PDK4 in cardiomyocytes by promoting its degradation by the mitochondrial Lon protease [80]. As estimated by the HIF-1α protein levels, which were markedly increased by MG-132, inhibition of the proteasome was effective in MDA-MB-231, PC-3, as well as L6 cells. However, MG-132 did not prevent the DCA-induced reduction of the PDK1 protein levels. Furthermore, MG-132 even appeared to promote loss of PDK1. MG-132 was previously shown to induce a paradoxical loss of cystic fibrosis transmembrane conductance regulator (CFTR) [81]. This effect was due to ubiquitination of immature forms of CFTR, which were detectable if the lysate sediment (which was insoluble in the Tris-based homogenization buffer) was solubilized in a buffer containing sodium dodecyl sulfate [81]. In our case, this explanation is unlikely, because we prepared whole-cell lysates from MG-132 experiments directly in the Laemmli buffer, which already contains sodium dodecyl sulfate, meaning that poorly soluble proteins were not lost during sample preparation.

Technical issues aside, it is nevertheless important to note that MG-132 may not lead to accumulation of the mature protein despite ubiquitination as observed for CFTR [81]. Importantly, MG-132 paradoxically promoted the loss of human anterior gradient 2 protein in lung cancer cells because it promoted the autophagic degradation of its polyubiquitinated form [82]. The loss of PDK1 protein in cells treated with MG-132 may therefore perhaps reflect an increase in autophagy. MG-132 may also attenuate the protein synthesis [83,84], which would contribute to the loss of PDK1. Thus, while our results do not support the involvement of the proteasome, its role in the DCA-induced suppression of PDK1 or PDK2 cannot be excluded and requires further investigation.

Notably, MG-132 appeared to potently increase the abundance of (ubiquitinated) phospho-PDK1 (Thr338) in L6 myotubes, which provides evidence that the phosphorylated PDK1 is degraded in the proteasome. The phosphorylation of Thr338 is mediated by PGK1 [70], a glycolytic enzyme that otherwise catalyzes the ATP-generating conversion of 1,3-diphosphoglycerate to 3-phosphoglycerate. The phosphorylation at Thr338 activates PDK1 [70], which might represent a compensation for the loss of PDK1. In L6 myotubes treated with MG-132, the abundance of phospho-PDK1 was reduced by DCA, which indirectly suggests that DCA might oppose the activation of PDK1 by phosphorylation at Thr338.

As well as MG-132, MG-262 (which inhibits proteasome and Lon protease), and calpain inhibitor III did not oppose the DCA-induced suppression of PDK1. We therefore examined whether the mitochondrial proteases CLPP and AFG3L2 [85] might be involved in proteolysis of PDK1. While substrates of these proteases require further characterization [86], nearly all PDC components seem to be substrates of CLPXP protease complex in a fungal model, suggesting a similar role for CLPP in human cells [87]. However, CLPP and AFG3L2 knockdown did not induce an accumulation of PDK1 in MDA-MB-231 and PC-3 cells under the basal conditions and it did not block the DCA-induced suppression of PDK1. Our results therefore indicate that PDK1 is likely not degraded by the proteasome, calpains, CLPP, or AFG3L2.

Finally, although investigation of cytotoxicity was not the aim of our study, it has to be noted that, as assessed by the Hoechst staining, DCA had only a minor effect on the proliferation of cancer cells, while metformin had no effect. Since DCA has been investigated as a possible therapeutic compound for various non-neoplastic conditions, including metabolic disorders [19], comparatively low cytotoxicity is not only expected, but also consistent with cancer cell data [53]. Similarly, our previous results showed that metformin had almost no effect on MDA-MB-231 cells if they were grown in the presence of normal glucose concentrations [50,65,66]. However, if they were exposed to low glucose concentrations, the effectiveness of metformin was markedly increased [50,65,66]. Moreover, a combination of DCA and metformin was shown to be markedly more cytotoxic than either treatment alone [53,55,88]. The effects of metabolic modulators, such as metformin and DCA, therefore vary depending on the metabolic state of cancer cells and/or presence of other pharmacological compounds. Taken together, while our study indicates that DCA would not be an efficient anti-cancer agent on its own, it does not oppose the notion that DCA may enhance the cytotoxic effects of metformin or cytotoxic drugs.

## 4. Materials and Methods

### 4.1. Materials

The rat skeletal muscle cell line L6, human breast adenocarcinoma cell line MDA-MB-231, and human prostate adenocarcinoma cell line PC-3 were obtained from ATCC (Manassas, VA, USA). Cell culture flasks and plates were from TPP (Trasadingen, Switzerlandt) or Sarstedt (Nümbrecht, Germany). RPMI-1640 was from Genaxxon bioscience (Ulm, Germany). MEMα with nucleosides (MEMα+), MEMα without nucleosides (MEMα−), fetal bovine serum (FBS), Pen Strep (5000 U/mL of penicillin and 5000 μg/mL of streptomycin), Fungizone (250 μg/mL of amphotericin B), Pierce BCA Protein Assay Kit, Pierce Enhanced Chemiluminescence (ECL) Western Blotting Substrate, PDK1 siRNA (4427038), scrambled siRNA (AM4611), transfection reagent Lipofectamine 2000, High-Capacity cDNA Reverse Transcription Kit, MicroAmp optical 96-well reaction plates, MicroAmp optical adhesive films, TaqMan Universal Master Mix and TaqMan gene expression assays for human HIF-1α (Hs00153153_m1), HIF-1β (Hs00231048_m1), PGK1 (4333765F), PDK1 (Hs01561847_m1), PDK2 (Hs00176865_m1), PDK3 (Hs00178440_m1), PDK4 (Hs01037712_m1), CLPP (Hs01548165_m1), AFG3L2 (Hs01064997_m1), VEGF (Hs00900054_m1), cyclophilin (PPIA) (Hs99999904_m1) and for rat PDK1 (Rn00587598_m1), PDK2 (Rn00446679_m1), PDK3 (Rn01424337_m1), PDK4 (Rn00585577_m1), and β-actin (Rn00667869_m1) were from Thermo Fisher Scientific (Waltham, MA, USA). HIF-1α siRNA (J-004018-10), HIF-1β siRNA pool (J-007207-06/-07/-08/-09), CLPP siRNA (L-005811-00-0005), AFG3L2 siRNA (L-005781-00-0005), and scrambled siRNA (D-001810-10-20) were from Dharmacon, Horizon (Cambridge, UK). The 4–12% Criterion XT Bis-Tris polyacrylamide gels, XT MES electrophoresis buffer, and goat anti-rabbit IgG-horseradish peroxidase conjugate were from Bio-Rad (Hercules, CA, USA). Amersham ECL Full-Range Rainbow Molecular Weight Markers were from GE Healthcare Life Sciences Cytiva (Marlborough, MA, USA). Polyvinylidene difluoride (PVDF) membrane was from Merck Millipore (Burlington, MA, USA). Primary antibodies are listed in Table 1. CP-BU NEW X-ray films were form AGFA HealthCare (Mortsel, Belgium). The RNeasy Plus Mini Kit was from Qiagen (Venlo, The Netherlands). Glutamine, HEPES, DCA, metformin, oxamate, NaCN, CoCl_2_, trifluoromethoxy carbonylcyanide phenylhydrazone (FCCP), transfection reagent X-tremeGENE 360, proteasome inhibitor MG-132, protease inhibitor cocktail, calpain inhibitor III, translation inhibitor puromycin, and Lactate assay kit were from Sigma-Aldrich (St. Louis, MO, USA). E.Z.N.A. HP Total RNA Isolation Kit was from Omega Bio-tek (Norcross, GA, USA). Fumarase Activity Colorimetric Assay Kit was from BioVision (Milpitas, CA, USA). Tetramethylrhodamine methyl ester (TMRM) and Hoechst 33342 were from Thermo Fisher Scientific (Waltham, MA, USA). All other reagents were from Sigma-Aldrich, unless specified otherwise.

### 4.2. Methods

#### 4.2.1. Cell Cultures and Experimental Conditions

MDA-MB-231 and PC-3 cells were cultured in RPMI-1640 medium supplemented with 1 g/l glucose, 2 mM glutamine, and 10% (*v/v*) FBS. Experiments were performed in RPMI-1640 without FBS (unless indicated otherwise). L6 myoblasts were maintained in MEMα medium with nucleosides (MEMα+) supplemented with 10% (*v/v*) FBS, 1% (*v/v*) PenStrep (50 U/mL of penicillin and 50 μg/mL of streptomycin), and 0.3% (*v/v*) Fungizone (0.75 μg/mL of amphotericin B). Differentiation of L6 myoblasts into myotubes was induced with MEMα+ medium supplemented with 2% (*v/v*) FBS, 1% (*v/v*) PenStrep, and 0.3% (*v/v*) Fungizone. Experiments were performed on the 7th to 10th day of differentiation of L6 myotubes in MEMα medium without nucleosides (MEMα−), FBS, or additives. Cells were cultured at 37 °C in humidified air with 5% (*v/v*) CO_2_. All experiments were performed in normoxia.

#### 4.2.2. Gene Silencing

MDA-MB-231 and PC-3 cells were seeded on 12-well plates overnight. Cells were transfected with PDK1 siRNA (10 nM), CLPP siRNA (10 nM), and/or AFG3L2 siRNA (10 nM), or with HIF-1α siRNA (5 nM) and HIF-1β siRNA (20 nM) or scrambled siRNA (5–20 nM). Lipofectamine 2000 was used as a transfection reagent for PDK1 siRNA, HIF-1α/1β siRNA, while X-tremeGENE 360 was used for CLPP siRNA and AFG3L2 siRNA, respectively. After 24 or 48 h, medium containing the transfection solution was removed and replaced with RPMI-1640 medium with 1 g/l glucose, 2 mM glutamine, and without FBS for a further 24 h of treatment with 10 mM DCA. Then, 48 or 72 h after transfection, cells were collected for qPCR and immunoblotting analysis.

#### 4.2.3. Quantitative Real-Time Polymerase Chain Reaction (qPCR)

Cells were lysed in the RLT Plus buffer supplemented with 1% (*v*/*v*) 2-mercaptoethanol. Total RNA, extracted with the RNeasy Plus Mini Kit or with E.Z.N.A. HP Total RNA Isolation Kit, was reverse transcribed with the High-Capacity cDNA Reverse Transcription Kit. qPCR was performed on an ABI PRISM SDS 7500 (Thermo Fisher Scientific (Waltham, MA, USA)) in a 96-well format, using TaqMan chemistry and TaqMan gene expression assays. The expression of target genes was normalized to the expression of human cyclophilin (PPIA) or rat β-actin (ACTB) for target genes in cancer and L6 myotubes, respectively. Standard quality controls were performed in line with the MIQE (minimum information for publication of quantitative real-time PCR experiments) Guidelines [89]. Expression levels were calculated by taking into account the efficiency of the PCR reaction calculated with the LinRegPCR software [90,91].

#### 4.2.4. Immunoblotting

Cells were lysed directly in 1× Laemmli buffer (62.5 mM Tris-HCl (pH 6.8), 2% (*w*/*v*) sodium dodecyl sulfate (SDS), 10% (*w*/*v*) glycerol, 5% (*v*/*v*) 2-mercaptoethanol, 0.002% (*w*/*v*) bromophenol blue) or the homogenization buffer (137 mM NaCl, 2.7 mM KCl, 1 mM MgCl_2_, 1% (*v*/*v*) Triton X-100, 10% (*w*/*v*) glycerol, 20 mM Tris (pH 7.8), 10 mM NaF, 1 mM EDTA, 0.5 mM Na_3_VO_4_, 1 mM phenylmethylsulfonyl fluoride, 1% (*v*/*v*) protease inhibitor cocktail). Insoluble components in cell lysates prepared in the homogenization buffer were removed by centrifugation (15 min, 12,000× *g*, at 4 °C). To these supernatants, used for protein content determination by Pierce BCA Protein Assay, 4× Laemmli buffer was added, and proteins were denaturated at 56 °C for 20 min. Samples were loaded on 4–12% Bis-Tris precast polyacrylamide gel and transferred to polyvinylidene difluoride membrane (PDVF) using the Criterion system. Proteins on the membranes were stained with Ponceau S (0.1% (*w*/*v*) in 5% (*v*/*v*) acetic acid) in order to evaluate sample loading and efficiency of the transfer. After blocking with 7.5% (*w*/*v*) low-fat dry milk in the Tris-buffered saline with Tween (TBST: 20 mM Tris, 150 mM NaCl, 0.02% (*v*/*v*) Tween 20, pH 7.5) for 1 h at room temperature, membranes were probed with primary antibodies at 4 °C overnight. Next, membranes were incubated with the horseradish peroxidase-conjugated secondary antibodies in 5% (*w*/*v*) low-fat dry milk in TBST for 1 h at room temperature. Blots were detected by the enhanced chemiluminescence (ECL) method using Pierce ECL Western Blotting Substrate on CP-BU NEW X-ray films or with Fusion FX (Vilber (Paris, France)). For blot quantifications, a GS800 Calibrated Densitometer (for blots on x-ray films) and Quantity One Software (BioRad (Hercules, California, USA)) were used.

#### 4.2.5. Lactate Measurement

Lactate was measured in cell medium in three experiments. First, MDA-MB-231 and PC-3 cells were treated with 10 mM oxamate, 10 mM DCA, 5 mM metformin, 5 mM NaCN, 250 μM CoCl_2_, metformin + NaCN, or metformin + CoCl_2_ in serum-free RPMI medium for 24 h. Second, MDA-MB-231 and PC3 cells were treated with 10 mM DCA and/or 5 mM metformin for 24 h. Third, after performing gene silencing of PDK1, MDA-MB-231 and PC-3 cells were treated with 5 mM metformin for 24 h. Upon completion of these 24-h treatments, medium samples were collected to measure the lactate concentrations using the enzymatic Lactate assay kit according to the manufacturer’s instructions. Results were normalized to the total protein concentration, which was determined using the Pierce BCA protein assay kit according to the manufacturer’s instructions.

#### 4.2.6. Measurement of Fumarase Activity

MDA-MB-231 and PC-3 cancer cells were treated with 10 mM oxamate, 10 mM DCA, 5 mM metformin, DMSO, or 0.5 μM FCCP in the RPMI medium supplemented with 1 g/L glucose, 2 mM glutamine, and 10% FBS for 24 h. The Fumarase Activity Colorimetric Assay Kit was used to determine fumarase activity following the manufacturer´s instructions. Results were normalized to the total protein concentration, which was determined using the Pierce BCA Protein Assay Kit following the manufacturer´s instructions.

#### 4.2.7. Measurement of the Mitochondrial Membrane Potential with TMRM

MDA-MB-231 and PC-3 cells were incubated with 200 nM tetramethylrhodamine methyl ester (TMRM) for 30 min at 37 °C. Cells were then detached and treated with 10 mM DCA, 10 mM oxamate, 10 mM NaCN, 5 μM oligomycin, oligomycin + NaCN, or 40 μM FCCP in suspension in PBS with 1 g/L glucose 10 mM HEPES, 2 mM glutamine, and 1% FBS for 40 min at room temperature. TMRM fluorescence was measured using a CyFlow Space flow cytometer (Sysmex Partec (Goerlitz, Germany)). Results were analyzed with FowJo software.

#### 4.2.8. Estimation of the DNA Content in MDA-MB-231 and PC-3 Cells

To indirectly assess the number of cells, we estimated the DNA content as described for MDA-MB-231 cells [50]. Briefly, the DNA content was estimated with a DNA assay based on the fluorescent DNA dye Hoechst 33342. [50]. After a 24-h treatment with DCA and other compounds (see Figure 1), cells were lysed in 0.03% solution (*w/v*) of SDS (in water) and lysate was transferred to a 96-well microplate and diluted (1:1) with Tris-NaCl buffer (50 mM Tris, 100 mM NaCl, pH 8.3) with 10 μg/mL Hoechst 33342. Samples were incubated for 15 min at room temperature and then Hoechst fluorescence was measured with a microplate reader (Victor (PerkinElmer, Waltham, Massachusetts, USA) or Tecan (Tecan, Männedorf, Switzerland)) using a 355 nm excitation filter and 460 nm emission filter.

#### 4.2.9. Statistical Analysis

Data are reported as mean ± standard deviation (SD) and were statistically evaluated using Prism version 6.0 (GraphPad Software, San Diego, CA, USA). Statistical differences were assessed with one-way ANOVA, followed by Dunnett’s or Bonferroni’s test. * *p* < 0.05 was considered statistically significant.

## 5. Conclusions

We found that DCA reduced the protein abundance of PDK1 in MDA-MB-231 and PC-3 cancer cells and the protein abundance of PDK1 and PDK2 in L6 myotubes. The reduction of the PDK1 levels can partially be explained by the DCA-stimulated degradation of HIF-1α in cancer cells, while HIF-1α does not seem to play a role in reducing the PDK1 protein levels in L6 myotubes. Our results suggest that post-transcriptional mechanisms are important for the suppression of PDK1 by DCA but also indicate that the involvement of the mTOR pathway, proteasome, CLPP, or AFG3L2 may not be required for this process. In conclusion, our study shows that DCA suppressed PDK in an isoform-dependent manner via transcriptional and post-transcriptional mechanisms. The differential response of PDK isoenzymes to DCA might be important for its pharmacological effects in different types of cells. 

## Figures and Tables

**Table 1:** Overview of the antibodies used for immunoblotting. Abbreviations: Ab—antibody, Mo—mouse, O/N—overnight, Rb—rabbit. Cell Signaling Technology (Danvers, MA, USA), Novus Biologicals (Centennial, CO, USA), Abcam (Cambridge, UK).

Primary Antibody Target	kDa	Primary Antibody					Secondary Antibody			
		Supplier	Cat. No.	Ab Host	Dilution	Time	Supplier	Cat. no.	Dilution	Time
phospho-AMPKα (Thr172)	62	Cell Signaling Technology	2535	Rb	1:1000	O/N	Bio-Rad	1706515	1:15,000	1 h
phospho-ACC (Ser79)	280	Cell Signaling Technology	3661	Rb	1:1000	O/N	Bio-Rad	1706515	1:15,000	1 h
phospho-4E-BP1 (Thr37/46)	15–20	Cell Signaling Technology	2855	Rb	1:1000	O/N	Bio-Rad	1706515	1:15,000	1 h
phospho-p70S6K (Thr389)	70,85	Cell Signaling Technology	9205	Rb	1:1000	O/N	Bio-Rad	1706515	1:10,000	1 h
PDK1	47	Cell Signaling Technology	3820	Rb	1:1000	O/N	Bio-Rad	1706515	1:8000	1 h
phospho-PDK1 (Thr338)	49	Signalway Antibody	11596	Rb	1:750	O/N	Bio-Rad	1706515	1:8000	1 h
PDK2	46	Novus Biologicals	NBP1-87307	Rb	1:800	O/N	Bio-Rad	1706515	1:8000	1 h
PDK3	47	Novus Biologicals	NBP1-32581	Rb	1:500	O/N	Bio-Rad	1706515	1:8000	1 h
phospho-PDHE1α (Ser293)	43	Abcam	ab92696	Rb	1:1000	O/N	Bio-Rad	1706515	1:50,000	1 h
HIF-1α	93	Novus Biologicals	NB100-449	Rb	1:1000	O/N	Bio-Rad	1706515	1:15,000	1 h
β-actin	42	Sigma-Aldrich	A5441	Mo	1:10,000	O/N	Bio-Rad	1706516	1:20,000	1 h

## Data Availability

Raw data of immunoblots (Supplement S4) and qPCR (Supplement S5) are available as Supplementary Materials to this paper. All other data are available without reservation upon reasonable request.

## Supplementary Materials

The following are available online at https://www.mdpi.com/article/10.3390/ijms22168610/s1, Figure S1 (supplementary figure to Figure 5): Inhibition of the mTOR pathway does not contribute to the DCA-induced suppression of PDK1, Figure S2 (supplementary figure to Figure 6): Inhibition of the proteasome or mitochondrial proteases does not prevent the DCA-induced suppression of PDK1 in cancer cells, Figure S3 (supplementary figure to Figure 7): DCA reduces the abundance of PDK1 and PDK2 in L6 myotubes despite upregulation of HIF-1α and inhibition of the proteasome, Supplement S4 (raw immunoblots), and Supplement S5 (qPCR data sets).

## Abbreviations

ACC	acetyl-CoA carboxylase
AFG3L2	AFG3-like protein 2
AMPK	AMP-activated protein kinase
ARNT	aryl hydrocarbon receptor nuclear translocator (aka HIF-1β)
CFTR	cystic fibrosis transmembrane conductance regulator
CLPP	caseinolytic mitochondrial matrix peptidase proteolytic subunit
DCA	dichloroacetate
FBS	foetal bovine serum
FCCP	trifluoromethoxy carbonylcyanide phenylhydrazone
HIF-1	hypoxia-inducible factor-1
mTOR	mammalian target of rapamycin
mTORC1	mammalian target of rapamycin complex 1
PBS	phosphate-buffered saline
PDC	pyruvate dehydrogenase complex
PDHE1α	pyruvate dehydrogenase E1 component subunit alpha
PDK	pyruvate dehydrogenase kinase
PGK1	phosphoglycerate kinase 1
p70S6K	p70 ribosomal protein S6 kinase
RPMI	Roswell Park Memorial Institute
TMRM	tetramethylrhodamine methyl ester
VEGF	vascular endothelial growth factor
4E-BP1	eukaryotic translation initiation factor 4E-binding protein 1
