# Supplementary material for: Suppression of Pyruvate Dehydrogenase Kinase by Dichloroacetate in Cancer and Skeletal Muscle Cells Is Isoform Specific and Partially Independent of HIF-1α
Source: Int J Mol Sci. 2021 Aug 10;22(16):8610. doi: 10.3390/ijms22168610 (PMC8395311; doi:10.3390/ijms22168610)
Supplement: Supplementary file 1 [file ijms-22-08610-s001.zip › ijms-1260980 figures.pdf]

Supplementary materials S1-S4

Suppression of pyruvate dehydrogenase kinase by dichloroacetate in cancer and skeletal muscle cells is isoform-specific and partially independent of HIF-1α

Nives Škorja Milić[1,2], Klemen Dolinar[1], Katarina Miš[1], Urška Matkovič[1,&], Maruša Bizjak[3,4], Mojca Pavlin[3,5], Matej Podbregar[1,6,7], and Sergej Pirkmajer [1,*]

[1] Institute of Pathophysiology, Faculty of Medicine, University of Ljubljana, Slovenia
[2] Institute of Anatomy, Faculty of Medicine, University of Ljubljana, Slovenia
[3] Group for nano and biotechnological applications, Faculty of Electrical Engineering, University of Ljubljana, Slovenia
[4] Pharmacy Institute, Faculty of Pharmacy, University of Ljubljana, Ljubljana, Slovenia
[5] Institute of Biophysics, Faculty of Medicine, University of Ljubljana, Ljubljana, Slovenia
[6] Department of Internal Medicine, Faculty of Medicine, University of Ljubljana, Slovenia
[7] Department for Internal Intensive care, General and Teaching Hospital Celje, Celje, Slovenia

[*] To whom correspondence should be addressed: sergej.pirkmajer@mf.uni-lj.si; Tel.: +386 1 543 7042

[&] Current address: Department of Experimental Oncology, Institute of Oncology Ljubljana, Ljubljana, Slovenia

Supplementary figures S1-S3

Supplementary Figure S1

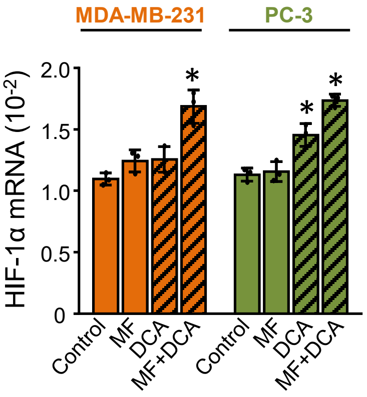

Supplementary figure S1 (supplementary figure to Figure 5): Inhibition of the mTOR pathway does not contribute to the DCA-induced suppression of PDK1. MDA-MB-231 and PC-3 cells were treated with 5 mM metformin (MF), 10 mM DCA, or both in serum-free RPMI medium for 24 hours. The mRNA expression of HIF-1α was measured with qPCR (endogenous control: cyclophilin). Results are means ± SD of one experiment performed in triplicate for each cell line (n = 3). * $p < 0.05$ vs. Control.

Supplementary Figure S2

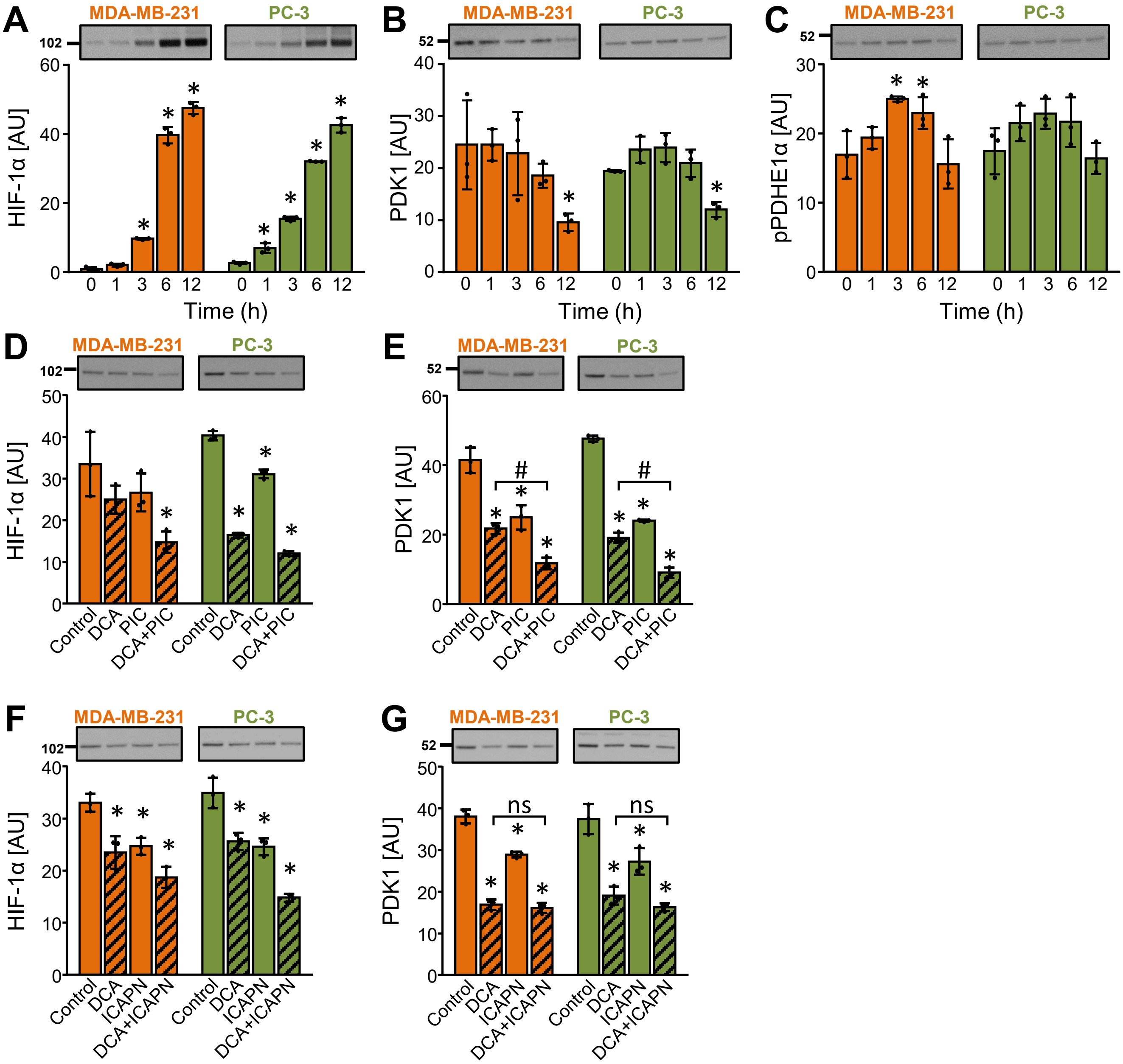

Supplementary figure S2 (supplementary figure to Figure 6): Inhibition of the proteasome or mitochondrial proteases does not prevent the DCA-induced suppression of PDK1 in cancer cells. (A–C) Cells were treated with MG-132 (10 µM) for 12 hours in serum-free RPMI medium. (D,E) Cells were treated with DCA (10 mM) and/or the protease inhibitor cocktail (PIC, 1:600) in the RPMI medium without serum (PC-3) or with 10% FBS (MDA-MB-231) for 24 hours. (F,G) Cells were treated with 9 µM (MDA-MB-231) or 45 µM (PC-3) calpain inhibitor III (ICAPN) in serum-free RPMI medium for 24 hours. DCA was added during the final 14 hours of treatment. The protein levels of HIF-1α, PDK1, and phospho-PDHE1α^{Ser293} (pPDHE1α) were determined with immunoblotting. Numbers next to the blots indicate molecular weight markers in kDa. Results are means ± SD of one experiment, performed in triplicate, for each cell line and each condition (n = 3). * $p < 0.05$ vs. 0 h or Control and # $p < 0.05$ between selected samples (as indicated).

Supplementary Figure S3

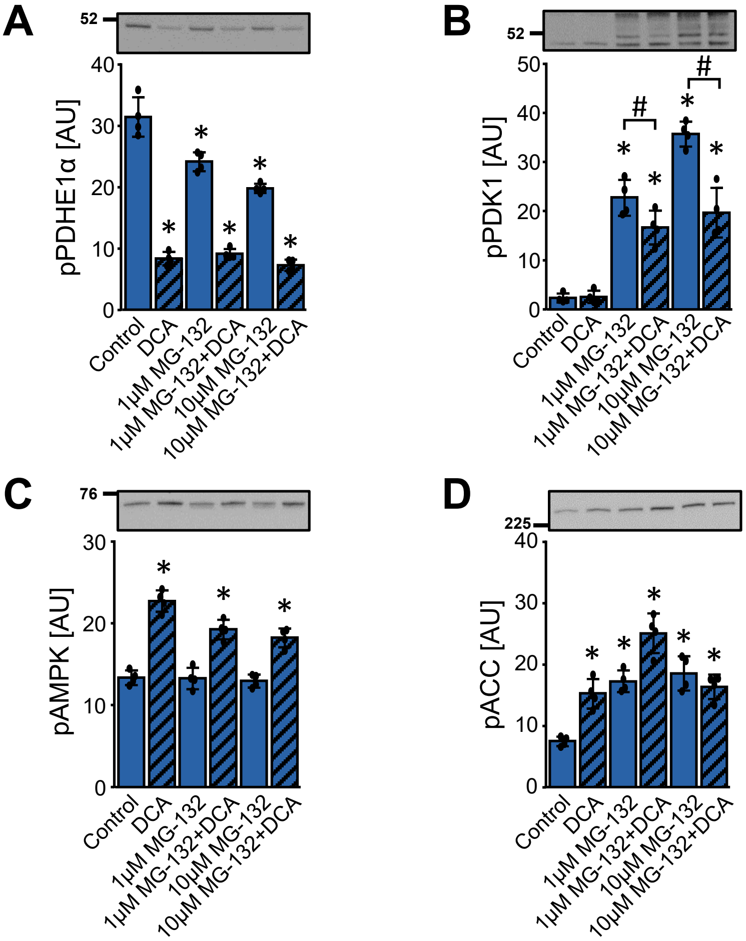

Supplementary figure S3 (supplementary figure to Figure 7): DCA reduces the abundance of PDK1 and PDK2 in L6 myotubes despite upregulation of HIF-1α and inhibition of the proteasome. The differentiated L6 cells (myotubes) were treated with MG-132 (1 or 10 μM) for 1 hour, which was followed by a 24-hour treatment with DCA (10 mM) and/or MG-132 (1 or 10 μM). Immunoblotting was used to estimate the abundance of (A) phospho-PDHE1α^{Ser293} (pPDHE1α), (B) phospho-PDK1^{Thr338} (pPDK1), (C) phospho-AMPKα^{Thr172} (pAMPK), and (D) phospho-ACC^{Ser79} (pACC). Numbers next to the blots indicate molecular weight markers in kDa. Results are means ± SD of one experiment in four replicates (n = 4). * $p < 0.05$ vs. Control and # $p < 0.05$ between selected samples (as indicated).

Supplement S4: Raw images

Immunoreactive bands were visualized on X-ray films using enhanced chemiluminescence and quantified using GS-800 Densitometer and Quantity One 1-D Analysis Software 4.6.8. (Bio-Rad, Hercules, CA, U.S.)

Loading and transfer were evaluated by Ponceau S (0.1% (w/v) in 5% (v/v) acetic acid) staining.

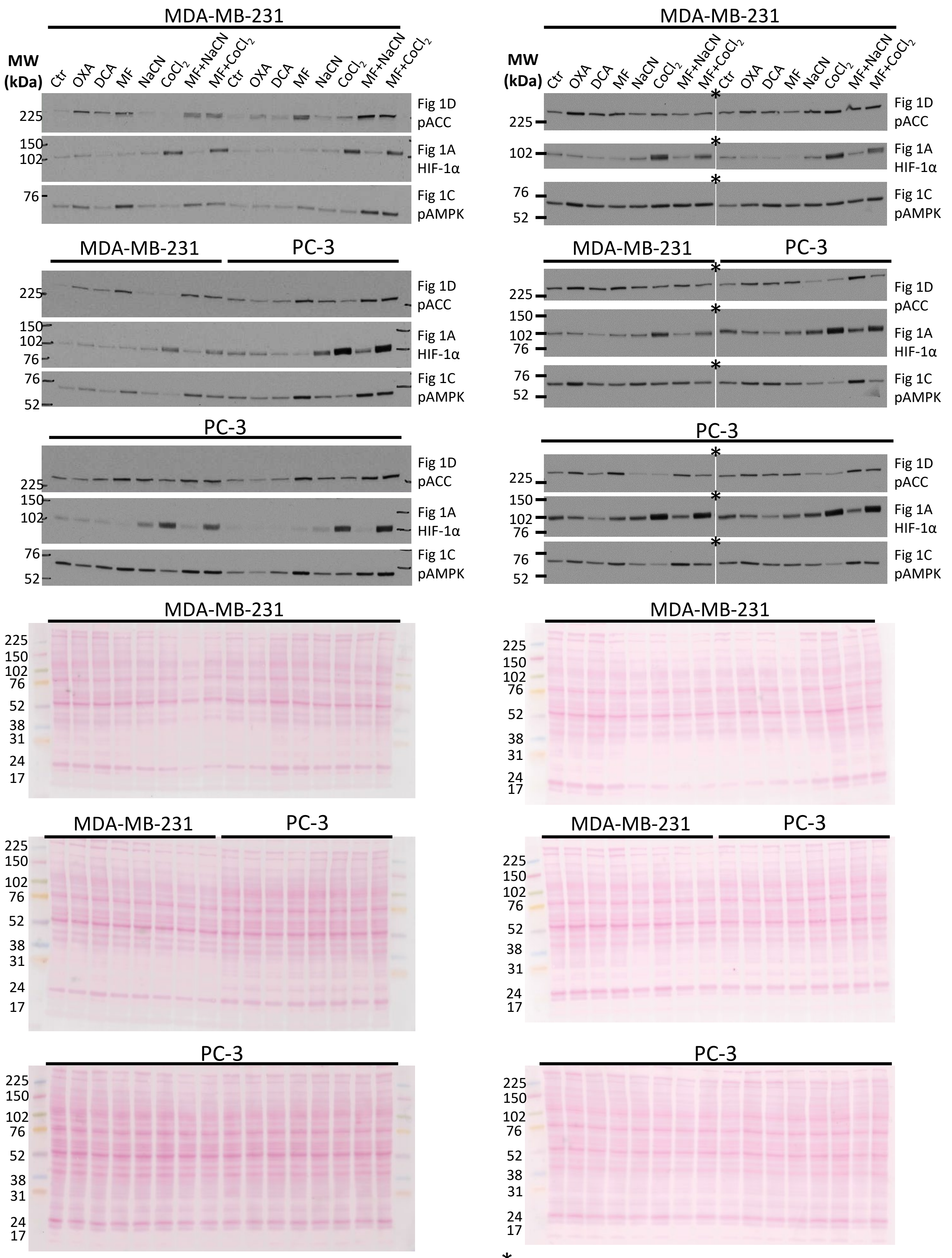

* Blots shown as originally taken on a Densitometer

Original uncropped blots presented in Figure 1 (1/2)

Immunoreactive bands were visualized with Fusion FX (Vilber) using enhanced chemiluminescence and quantified using Quantity One 1-D Analysis Software 4.6.8. (Bio-Rad, Hercules, CA, U.S.)

Loading and transfer were evaluated by Ponceau S (0.1% (w/v) in 5% (v/v) acetic acid) staining.

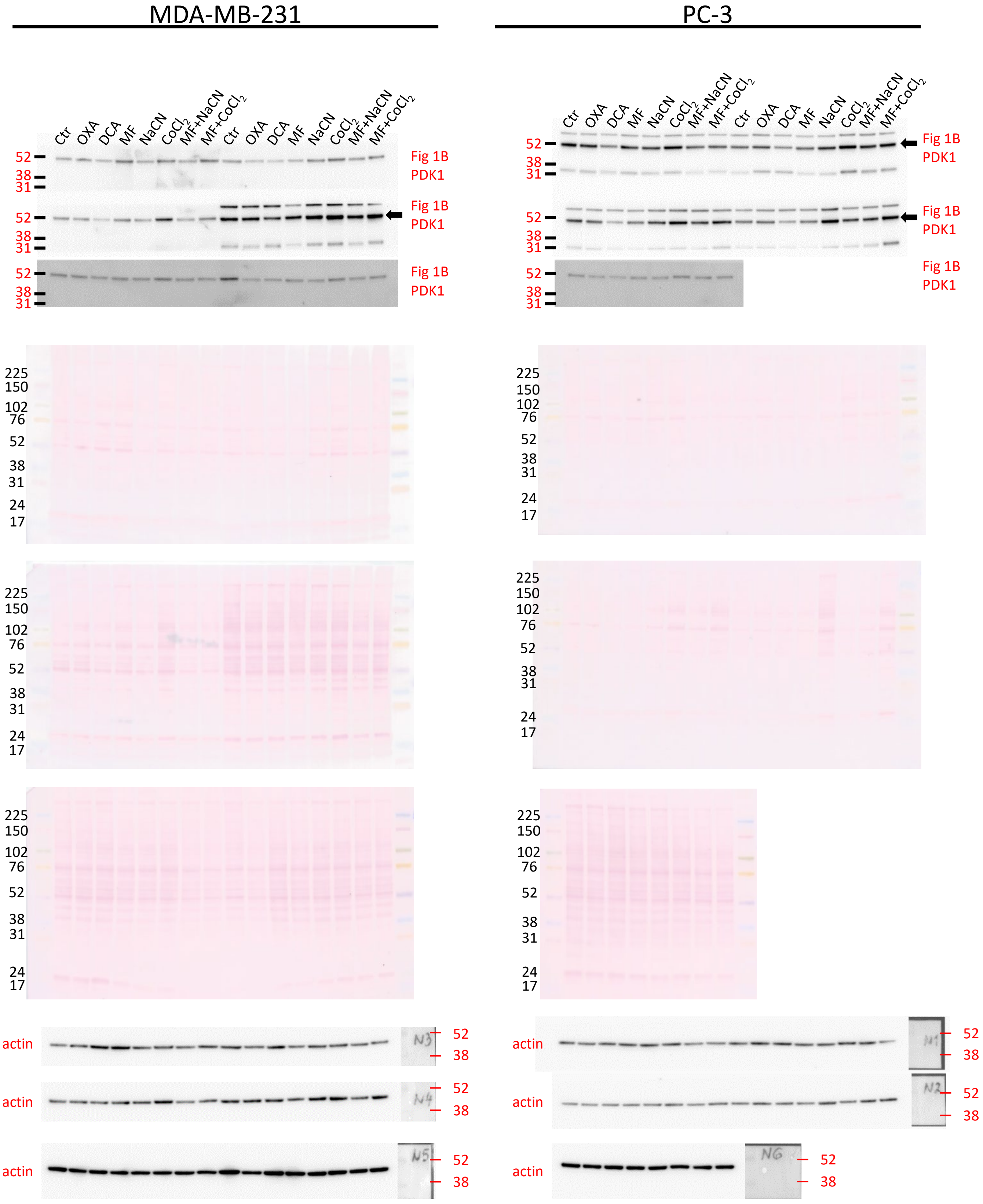

Original uncropped blots presented in Figure 1 (2/2)

Immunoreactive bands were visualized on X-ray films or with Fusion FX (Vilber) using enhanced chemiluminescence and quantified using GS-800 Densitometer and Quantity One 1-D Analysis Software 4.6.8. (Bio-Rad, Hercules, CA, U.S.)

Loading and transfer were evaluated by Ponceau S (0.1% (w/v) in 5% (v/v) acetic acid) staining.

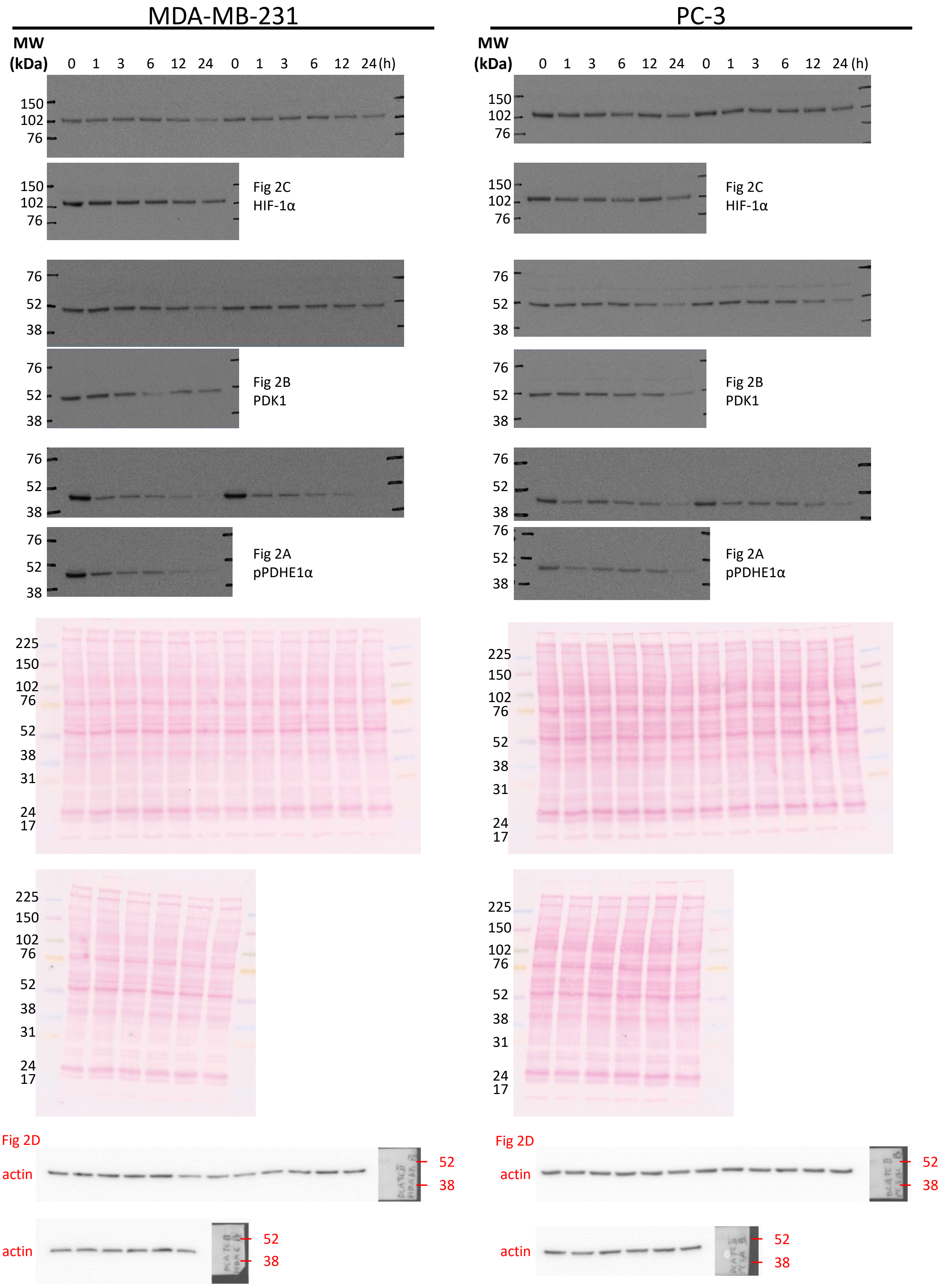

Original uncropped blots presented in Figure 2

Immunoreactive bands were visualized on X-ray films using enhanced chemiluminescence and quantified using GS-800 Densitometer and Quantity One 1-D Analysis Software 4.6.8. (Bio-Rad, Hercules, CA, U.S.)

Loading and transfer were evaluated by Ponceau S (0.1% (w/v) in 5% (v/v) acetic acid) staining.

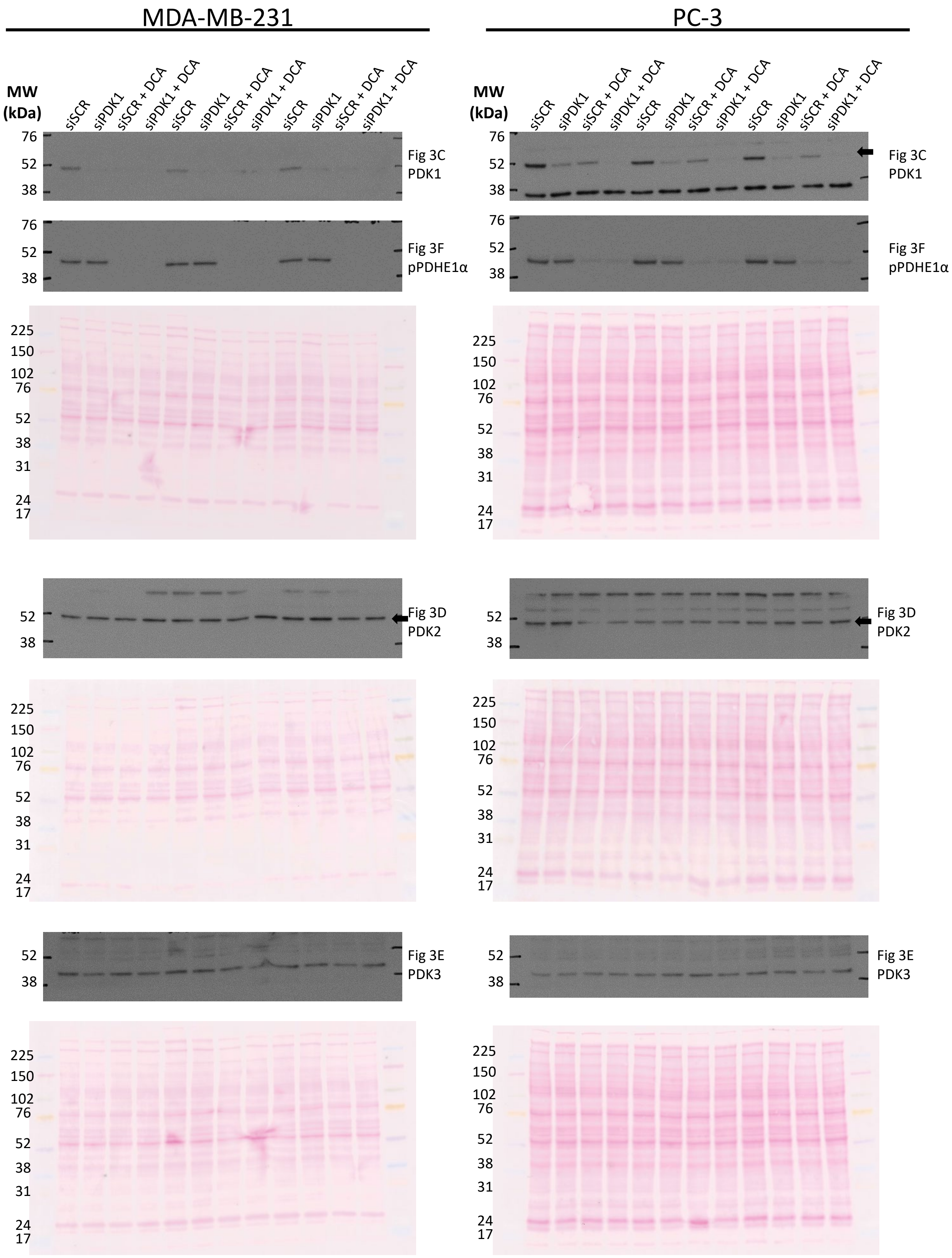

Original uncropped blots presented in Figure 3

Immunoreactive bands were visualized on X-ray films or with Fusion FX (Vilber) using enhanced chemiluminescence and quantified using GS-800 Densitometer and Quantity One 1-D Analysis Software 4.6.8. (Bio-Rad, Hercules, CA, U.S.)

Loading and transfer were evaluated by Ponceau S (0.1% (w/v) in 5% (v/v) acetic acid) staining.

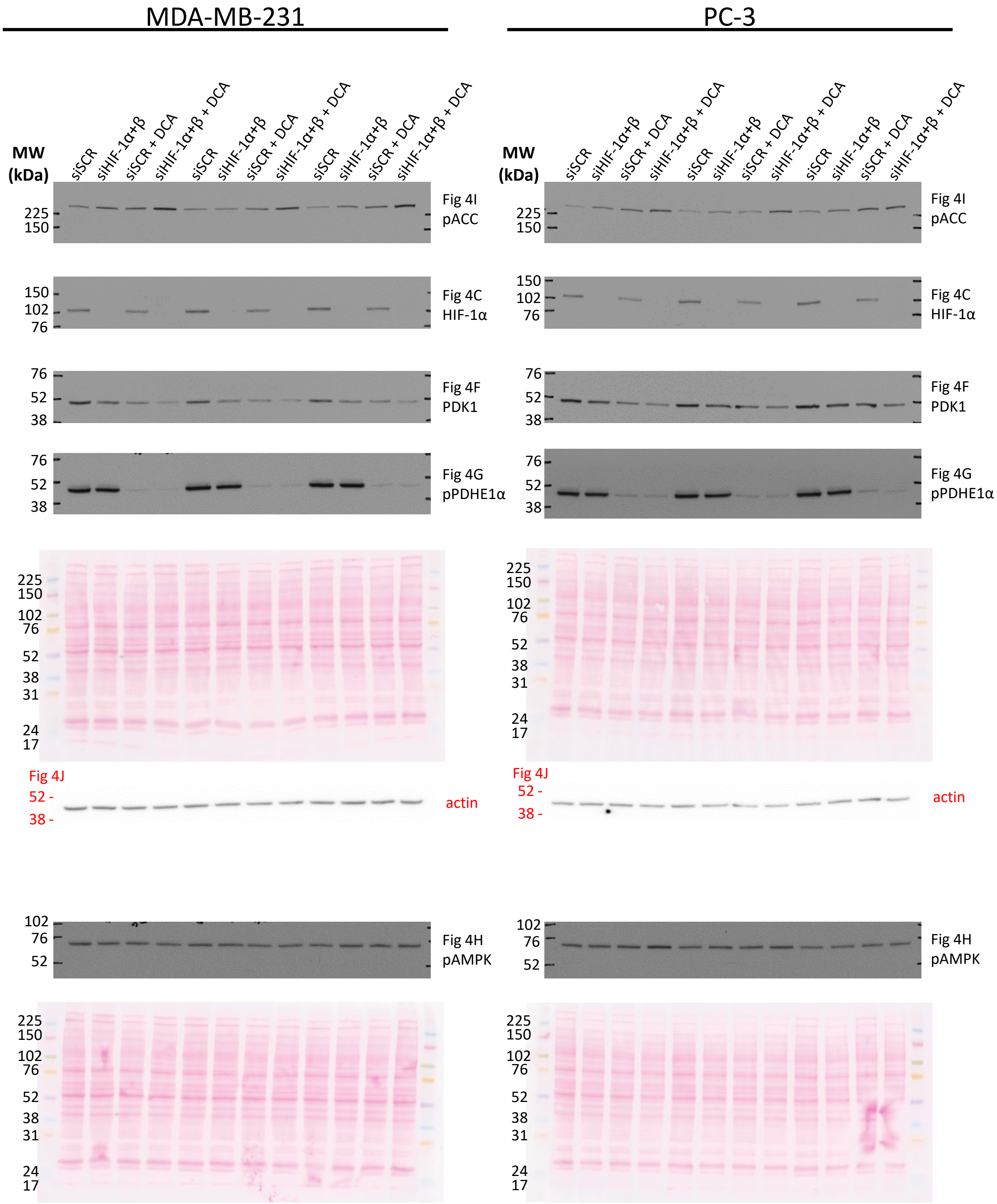

Original uncropped blots presented in Figure 4

Immunoreactive bands were visualized on X-ray films or with Fusion FX (Vilber) using enhanced chemiluminescence and quantified using GS-800 Densitometer and Quantity One 1-D Analysis Software 4.6.8. (Bio-Rad, Hercules, CA, U.S.)

Loading and transfer were evaluated by Ponceau S (0.1% (w/v) in 5% (v/v) acetic acid) staining.

MDA-MB-231

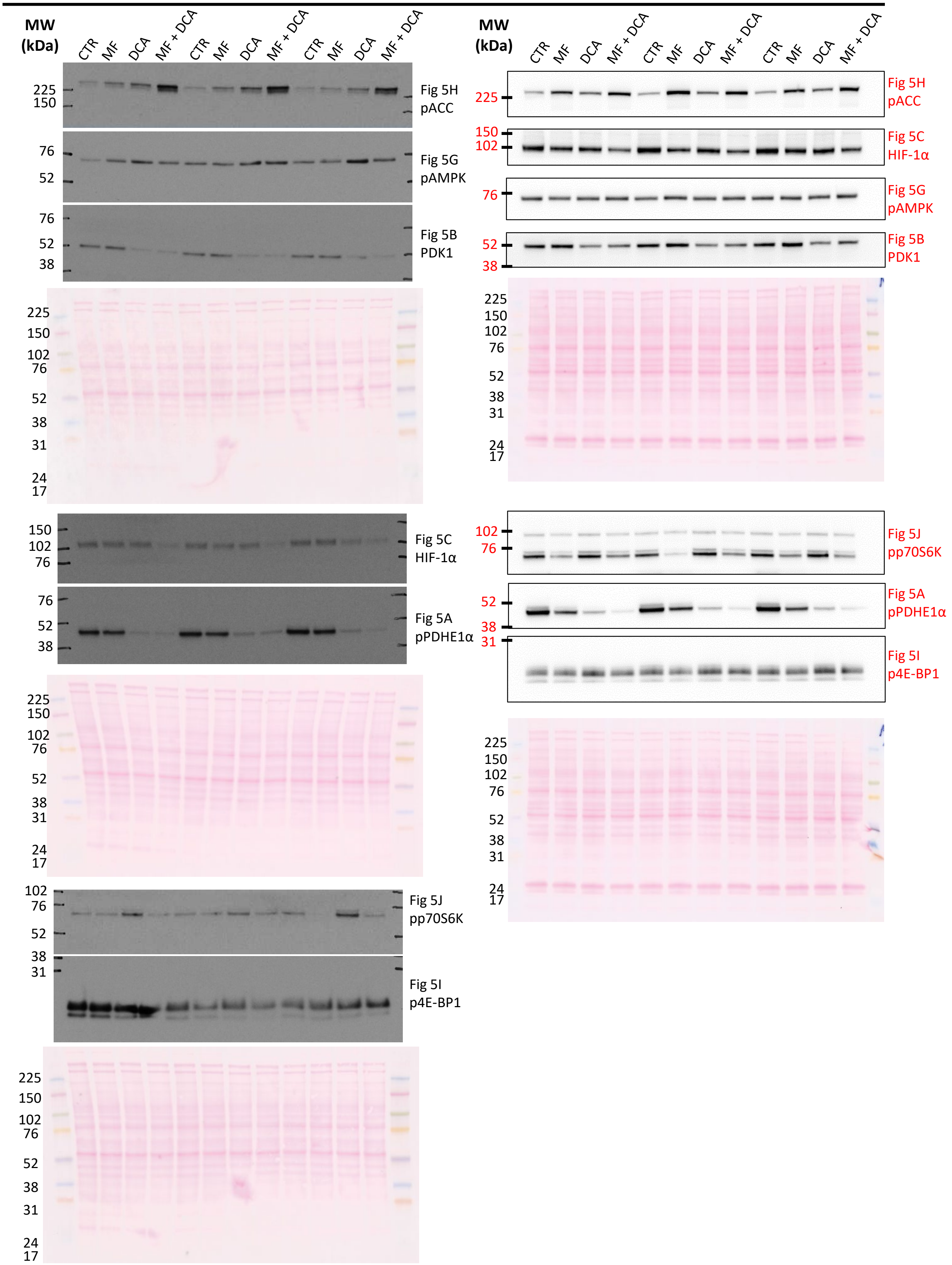

Original uncropped blots presented in Figure 5 (1/2)

Immunoreactive bands were visualized on X-ray films using enhanced chemiluminescence and quantified using GS-800 Densitometer and Quantity One 1-D Analysis Software 4.6.8. (Bio-Rad, Hercules, CA, U.S.)

Loading and transfer were evaluated by Ponceau S (0.1% (w/v) in 5% (v/v) acetic acid) staining.

PC-3

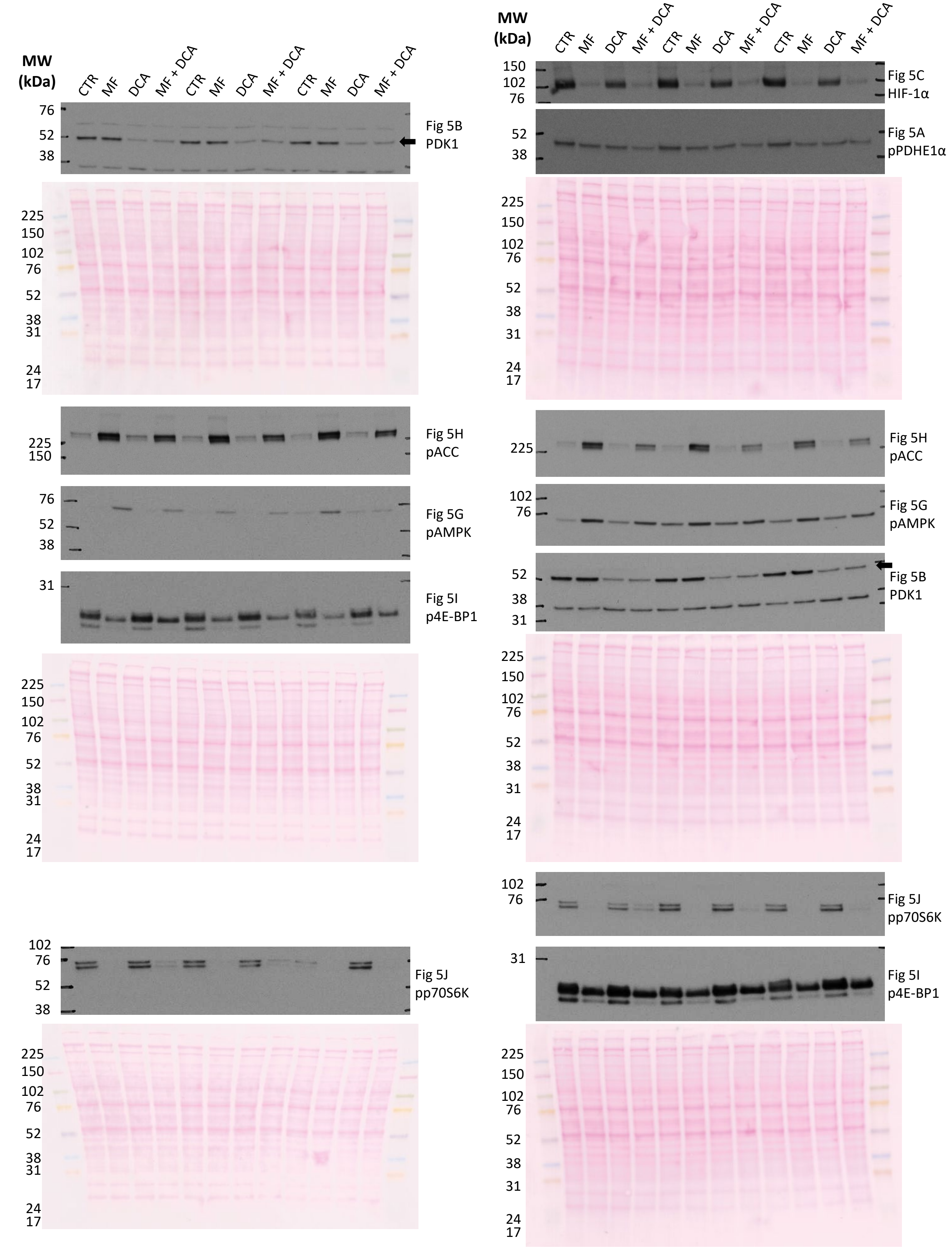

Original uncropped blots presented in Figure 5 (2/2)

Immunoreactive bands were visualized on X-ray films using enhanced chemiluminescence and quantified using GS-800 Densitometer and Quantity One 1-D Analysis Software 4.6.8. (Bio-Rad, Hercules, CA, U.S.)

Loading and transfer were evaluated by Ponceau S (0.1% (w/v) in 5% (v/v) acetic acid) staining.

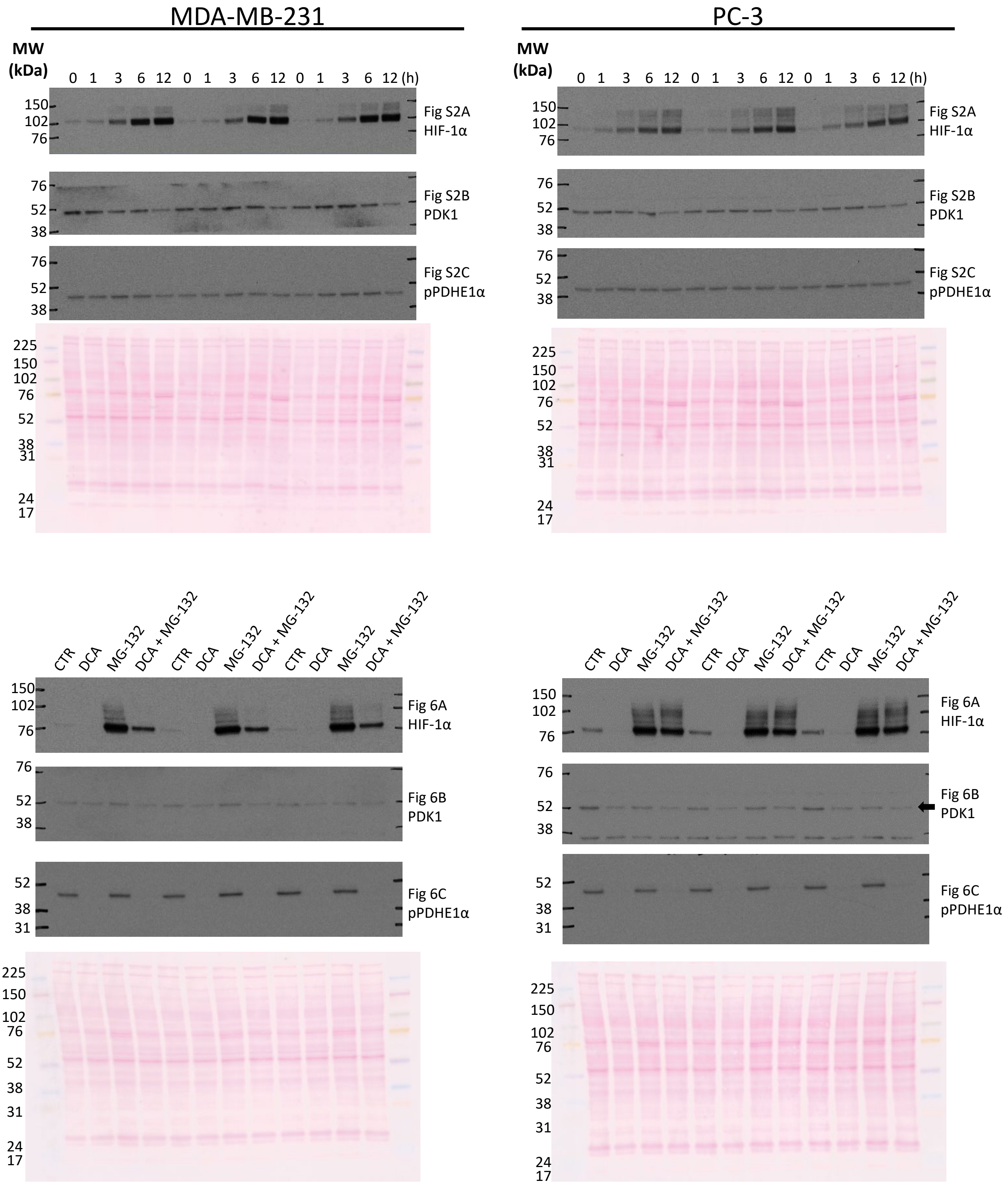

Original uncropped blots presented in Figure 6 and S2 (1/3)

Immunoreactive bands were visualized on X-ray films using enhanced chemiluminescence and quantified using GS-800 Densitometer and Quantity One 1-D Analysis Software 4.6.8. (Bio-Rad, Hercules, CA, U.S.)

Loading and transfer were evaluated by Ponceau S (0.1% (w/v) in 5% (v/v) acetic acid) staining.

MDA-MB-231

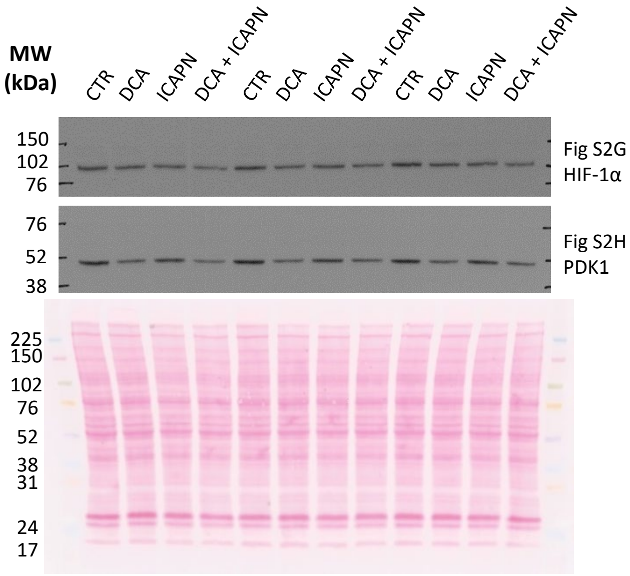

PC-3

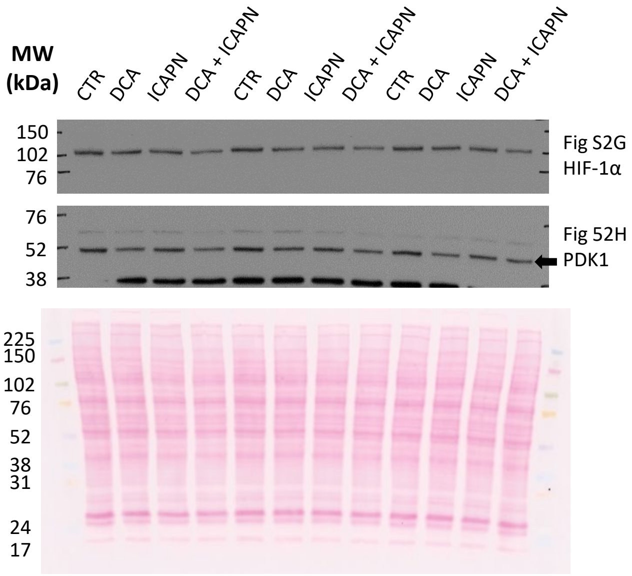

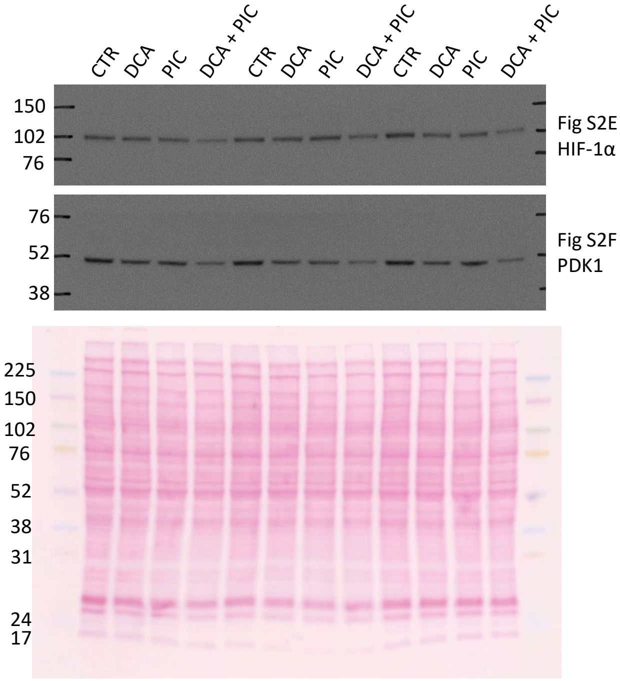

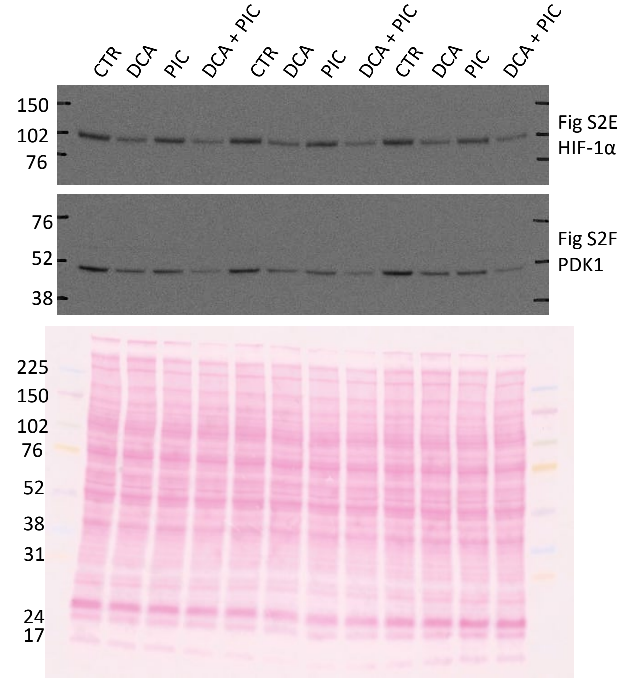

Original uncropped blots presented in Figure 6 and S2 (2/3)

Immunoreactive bands were visualized on X-ray films or with Fusion FX (Vilber) using enhanced chemiluminescence and quantified using GS-800 Densitometer and Quantity One 1-D Analysis Software 4.6.8. (Bio-Rad, Hercules, CA, U.S.)

Loading and transfer were evaluated by Ponceau S (0.1% (w/v) in 5% (v/v) acetic acid) staining.

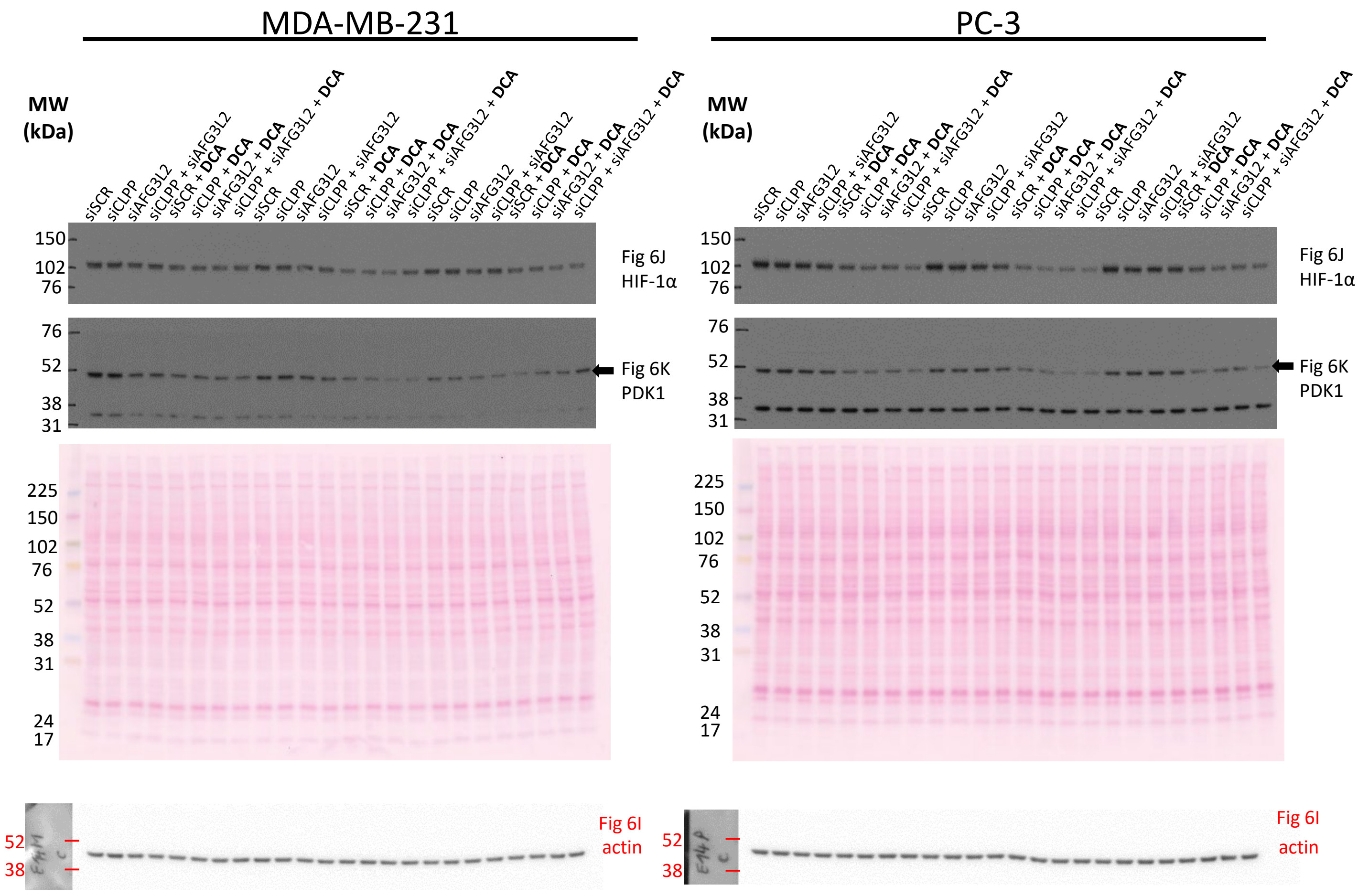

Original uncropped blots presented in Figure 6 (3/3)

Immunoreactive bands were visualized on X-ray films or with Fusion FX (Vilber) using enhanced chemiluminescence and quantified using GS-800 Densitometer and Quantity One 1-D Analysis Software 4.6.8. (Bio-Rad, Hercules, CA, U.S.)

Loading and transfer were evaluated by Ponceau S (0.1% (w/v) in 5% (v/v) acetic acid) staining.

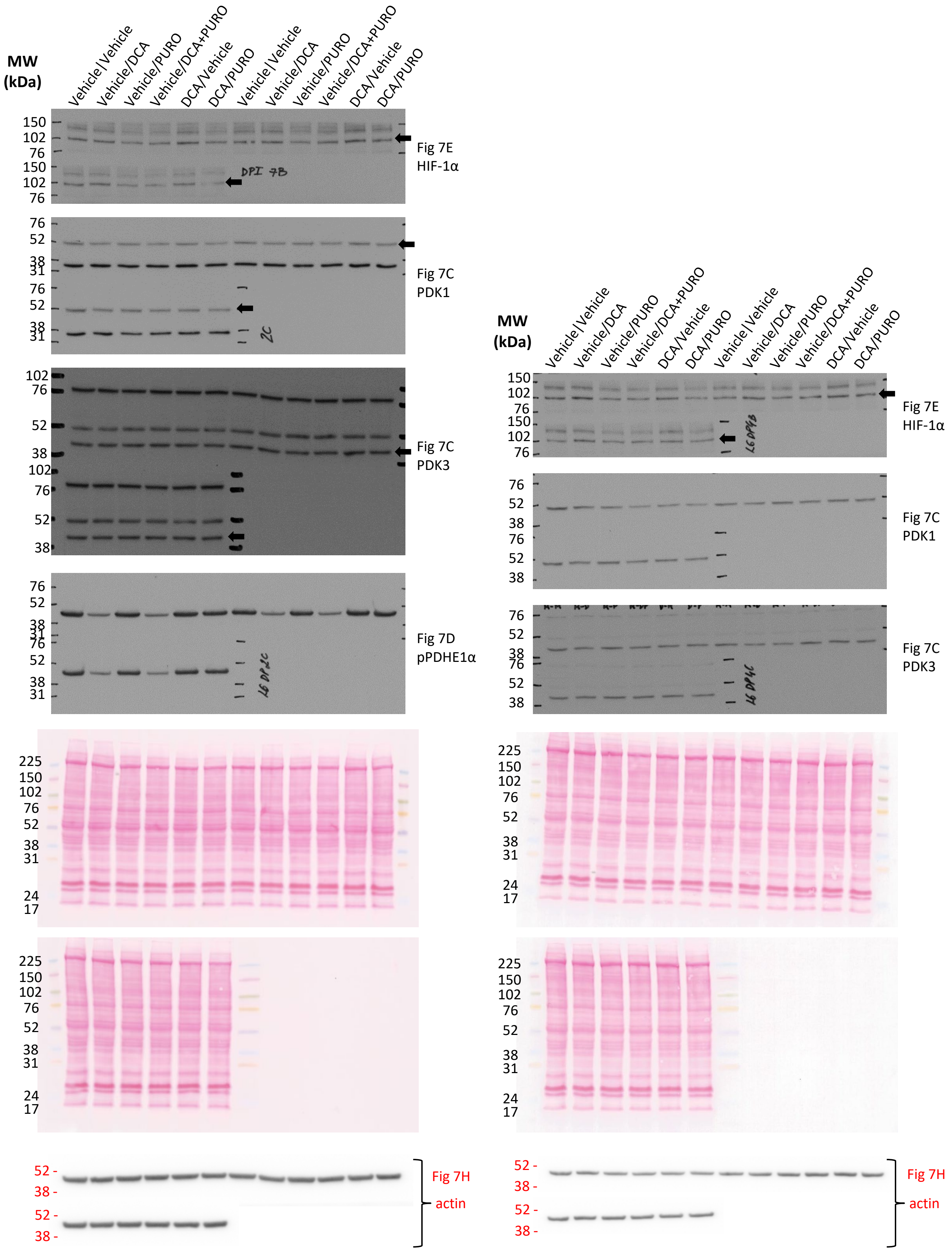

Original uncropped blots presented in Figure 7 and S3 (1/4)

Immunoreactive bands were visualized on X-ray films using enhanced chemiluminescence and quantified using GS-800 Densitometer and Quantity One 1-D Analysis Software 4.6.8. (Bio-Rad, Hercules, CA, U.S.)

Loading and transfer were evaluated by Ponceau S (0.1% (w/v) in 5% (v/v) acetic acid) staining.

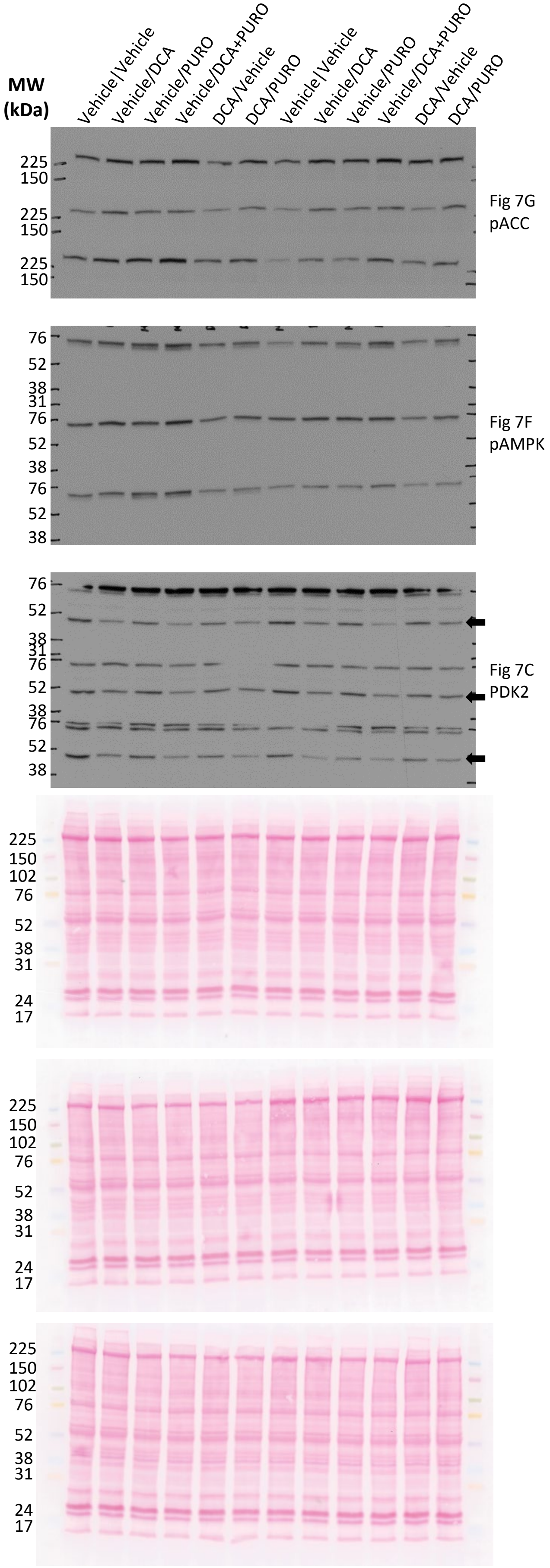

Original uncropped blots presented in Figure 7 and S3 (2/4)

Immunoreactive bands were visualized on X-ray films using enhanced chemiluminescence and quantified using GS-800 Densitometer and Quantity One 1-D Analysis Software 4.6.8. (Bio-Rad, Hercules, CA, U.S.)

Loading and transfer were evaluated by Ponceau S (0.1% (w/v) in 5% (v/v) acetic acid) staining.

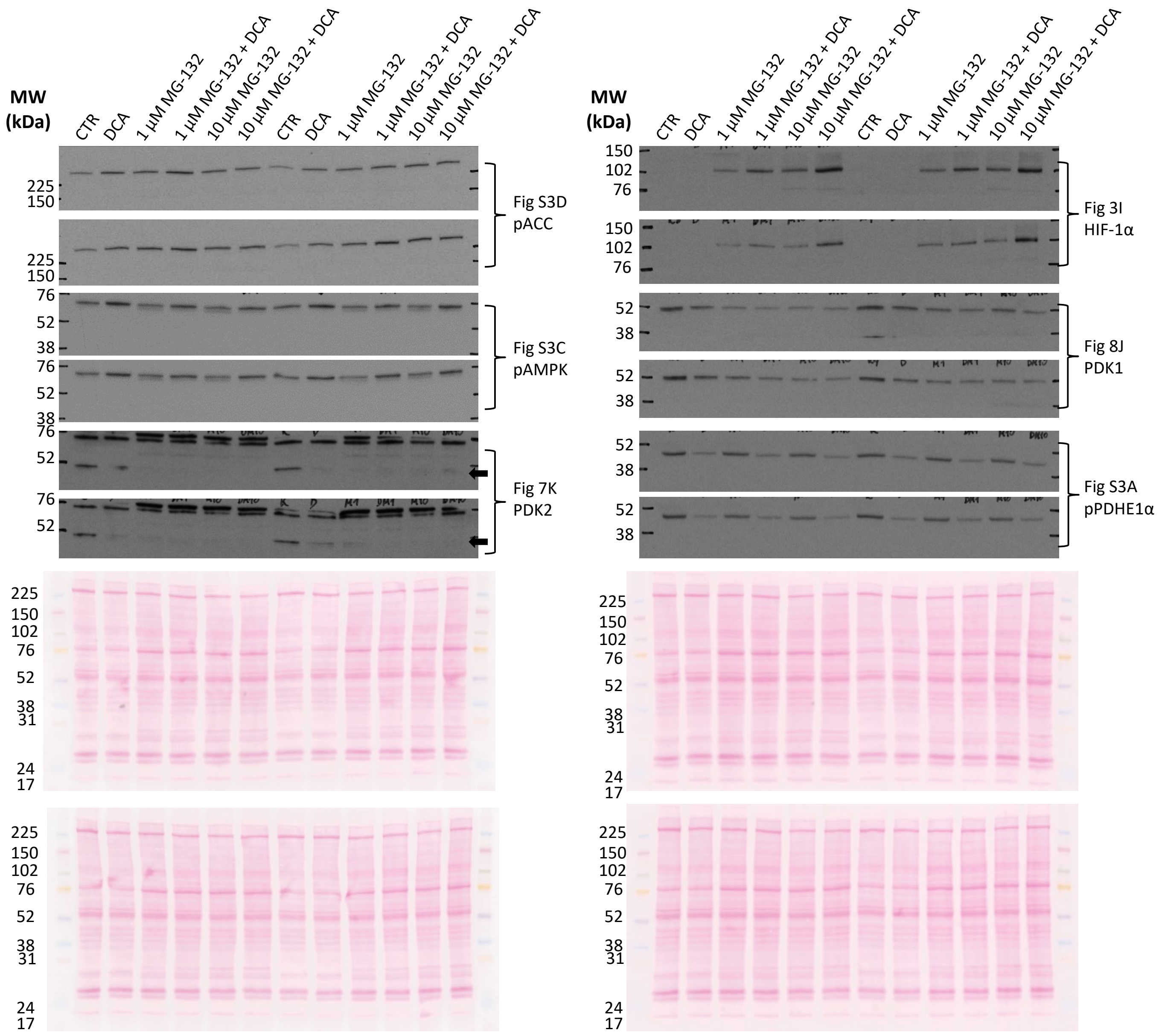

Original uncropped blots presented in Figure 7 and S3 (3/4)

Immunoreactive bands were visualized on X-ray films or with Fusion FX (Vilber) using enhanced chemiluminescence and quantified using GS-800 Densitometer and Quantity One 1-D Analysis Software 4.6.8. (Bio-Rad, Hercules, CA, U.S.)

Loading and transfer were evaluated by Ponceau S (0.1% (w/v) in 5% (v/v) acetic acid) staining.

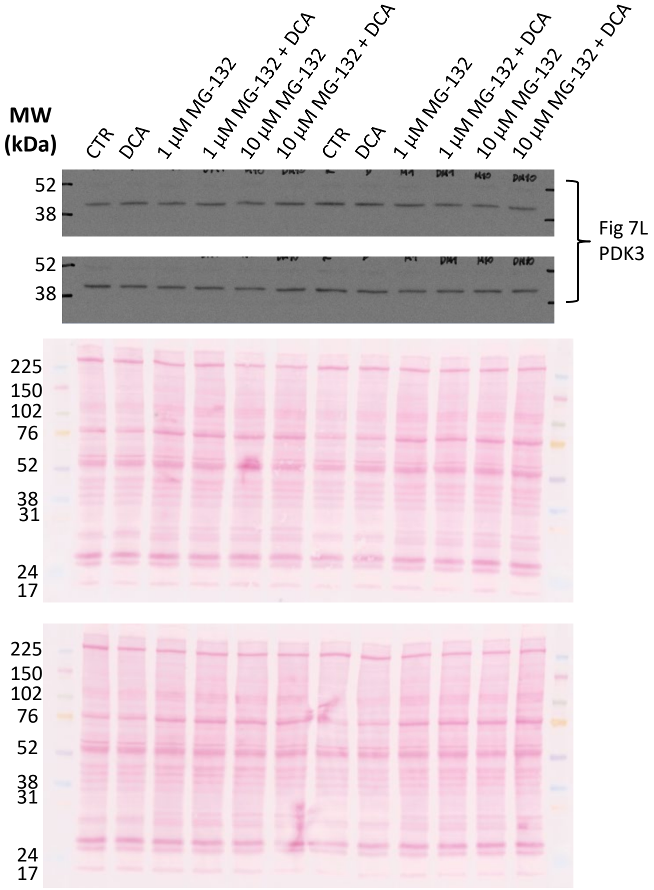

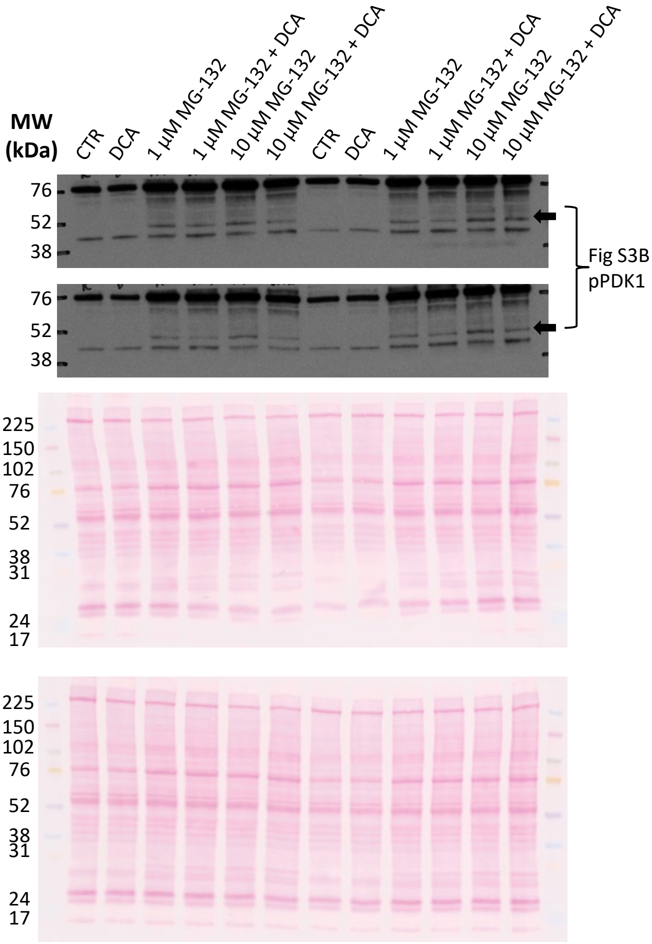

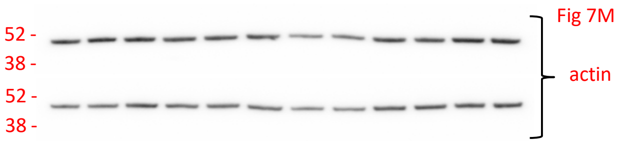

Original uncropped blots presented in Figure 7 and S3 (4/4)
